# Large-scale phosphoproteomics reveals activation of the MAPK/GADD45β/P38 axis and cell cycle inhibition in response to BMP9 and BMP10 stimulation in endothelial cells

**DOI:** 10.1186/s12964-024-01486-0

**Published:** 2024-03-04

**Authors:** Mohammad Al Tarrass, Lucid Belmudes, Dzenis Koça, Valentin Azemard, Hequn Liu, Tala Al Tabosh, Delphine Ciais, Agnès Desroches-Castan, Christophe Battail, Yohann Couté, Claire Bouvard, Sabine Bailly

**Affiliations:** 1 Biosanté Unit U1292, Grenoble Alpes University, CEA, Grenoble, 38000 France; 2 Grenoble Alpes University, CEA, INSERM, UA13 BGE, CNRS, CEA, FR2048, Grenoble, France; 3 Present address: Université Côte d’Azur, CNRS, INSERM, iBV, Nice, France

**Keywords:** BMP9, BMP10, Endothelial cells, Signaling, Phosphoproteomics, MAPK, Proliferation

## Abstract

**Background:**

BMP9 and BMP10 are two major regulators of vascular homeostasis. These two ligands bind with high affinity to the endothelial type I kinase receptor ALK1, together with a type II receptor, leading to the direct phosphorylation of the SMAD transcription factors. Apart from this canonical pathway, little is known. Interestingly, mutations in this signaling pathway have been identified in two rare cardiovascular diseases, hereditary hemorrhagic telangiectasia and pulmonary arterial hypertension.

**Methods:**

To get an overview of the signaling pathways modulated by BMP9 and BMP10 stimulation in endothelial cells, we employed an unbiased phosphoproteomic-based strategy. Identified phosphosites were validated by western blot analysis and regulated targets by RT-qPCR. Cell cycle analysis was analyzed by flow cytometry.

**Results:**

Large-scale phosphoproteomics revealed that BMP9 and BMP10 treatment induced a very similar phosphoproteomic profile. These BMPs activated a non-canonical transcriptional SMAD-dependent MAPK pathway (MEKK4/P38). We were able to validate this signaling pathway and demonstrated that this activation required the expression of the protein GADD45β. In turn, activated P38 phosphorylated the heat shock protein HSP27 and the endocytosis protein Eps15 (EGF receptor pathway substrate), and regulated the expression of specific genes (E-selectin, hyaluronan synthase 2 and cyclooxygenase 2). This study also highlighted the modulation in phosphorylation of proteins involved in transcriptional regulation (phosphorylation of the endothelial transcription factor ERG) and cell cycle inhibition (CDK4/6 pathway). Accordingly, we found that BMP10 induced a G1 cell cycle arrest and inhibited the mRNA expression of *E2F2*, *cyclinD1* and *cyclinA1*.

**Conclusions:**

Overall, our phosphoproteomic screen identified numerous proteins whose phosphorylation state is impacted by BMP9 and BMP10 treatment, paving the way for a better understanding of the molecular mechanisms regulated by BMP signaling in vascular diseases.

**Supplementary Information:**

The online version contains supplementary material available at 10.1186/s12964-024-01486-0.

## Background

BMPs (Bone morphogenetic proteins) belong to the TGF-β (Transforming growth factor) superfamily, which comprises 33 cytokines in mammals, together with GDFs (Growth and differentiation factors), activins, inhibins and nodal. These cytokines are involved in the regulation of many biological processes, including differentiation, morphogenesis, proliferation and tissue homeostasis [1]. Among BMPs, BMP9 and BMP10 have been shown to play a key specific role in vascular homeostasis by binding to their high affinity receptor ALK1, which is mainly expressed on endothelial cells (ECs) [2]. BMP9 and BMP10 bind to a signaling complex composed of two type I (ALK1) and two type II (BMPRII, ActRIIA or ActRIIB) serine/threonine kinase transmembrane receptors, with BMPRII being much more expressed on ECs compared to the two other type II receptors. Following ligand binding, the type II receptor phosphorylates the GS (glycine/serine) domain of ALK1 [2]. Consequently, activated ALK1 phosphorylates the C-terminus of SMAD1 and SMAD5, allowing the recruitment of the regulatory co-SMAD, SMAD4. This trimeric SMAD complex subsequently translocates to the nucleus, where it binds to the promoters of many target genes with the assistance of other transcriptional regulators, thereby regulating their expression levels. This pathway is known as the canonical SMAD signaling pathway [3].

Mutations in this signaling pathway are responsible of two rare vascular diseases. Hereditary hemorrhagic telangiectasia (HHT) is caused by heterozygous loss-of-function mutations in the genes coding for ALK1 (*ACVRL1*), the co-receptor endoglin (*ENG*), and, less frequently, in the transcription factor *SMAD4*. On the other hand, in heritable pulmonary arterial hypertension (HPAH), the majority of cases involve mutations in the gene coding for the type II receptor, *BMPR2*, with only very rare cases caused by *ACVRL1* mutations [4]. Additionally, mutations in the genes encoding the two ligands BMP9 (*GDF2*) and *BMP10* have been recently described in HPAH and, more rarely, in HHT [2].

Over the past two decades, various experimental findings have indicated that ligands belonging to the TGF-β family can activate, in addition to the well-studied canonical SMAD signaling pathway, non-canonical signaling pathways [5–7]. These include the mitogen activated protein kinase (MAPK) cascades, involving P38, c-Jun N-terminal kinases (JNK), or extracellular signal-regulated kinases 1 (ERK1) and ERK2, but also the PI3K-AKT pathway and Rho-like GTPases. Much less is known concerning the non-canonical signaling pathways regulated by BMPs [8]. These pathways seem to be more cell context-dependent than the canonical SMAD pathway and have not been extensively studied in ECs in response to BMPs. In this work, we investigated signaling pathways modulated in ECs by stimulation with BMP9 or BMP10 using high resolution mass spectrometry (MS)-based phosphoproteomics.

## Methods

### Cell culture

Human umbilical vein endothelial cells (HUVECs) were purchased from Lonza and cultured in endothelial basal medium-2 (EBM-2) supplemented with EGM-2MV SingleQuot additives (fetal bovine serum (5% FBS), hEGF, hydrocortisone, VEGF, hFGF-B, R3-IGF-1, ascorbic acid, GA-1000, and heparin; Lonza). Murine NIH-3T3 fibroblasts were maintained in high glucose, sodium pyruvate and GlutaMAX-supplemented Dulbecco’s modified Eagle medium (DMEM; Gibco) with 10% FBS (Biosera) and 1% Penicillin/Streptomycin (Gibco). Cells were incubated at 37 ℃ in a humidified atmosphere containing 5% CO2.

### Workflow for phosphoproteome sample preparation

#### Stimulation and cell lysis

HUVECs were serum-starved for 6 hours (h) in EBM-2, after which they were stimulated or not for 30 minutes (min) with 10 ng/mL of BMP9 or BMP10. Stimulations were repeated two times with 3 biological replicates/condition in the first experiment and 2 biological replicates/condition in the second experiment generating 5 biological replicates/condition. Cells were then lysed on ice with urea lysis buffer (8 M Urea, 50 mM Tris–HCl pH 8, 75 mM NaCl, 1 mM EDTA) supplemented with protease inhibitor cocktail (P8340) and phosphatase inhibitor cocktails 2 (P5726) and 3 (P0044) purchased from Sigma. Protein concentrations were determined using a µ-BCA assay kit (Thermo Fisher Scientific).

#### Sample preparation

1 mg proteins from each sample was used to prepare samples for nLC-MS/MS analyses. Extracted proteins were reduced using 20 mM of dithiothreitol (DTT) for 1 h at 37 °C before alkylation with 55 mM of iodoacetamide for 1 h at 37 °C in the dark. Samples were then diluted to ½ using ammonium bicarbonate and digested with LysC (Promega) at a ratio of 1:200 for 4 h at 37 °C. Then they were diluted again to ¼ and digested overnight at 37 °C with sequencing grade-modified trypsin (Promega) at a ratio of 1:50. Resulting peptides were purified by C18 reverse phase chromatography (Sep-Pak C18, Waters) before drying down.

Peptides were then labelled using an isobaric labelling-based approach, relying on tandem mass tags (TMT) [9] using the 16plex TMTpro isobaric Label Reagent kit (ThermoFisher Scientific) before mixing equivalent amounts and desalting using C18 reverse phase chromatography (Sep-Pak C18, Waters). Labeled peptides were split into three different aliquots: ~ 60 µg for proteome wide analysis, and 2 aliquots of ~ 7 mg for generating 2 technical replicates of phosphopeptide enrichment. Phosphopeptide enrichment was performed using titanium dioxide beads (TitanSphere, GL Sciences, Inc.) as previously described [10] before purification using C18 reverse phase chromatography (Marco SpinColumns, Harvard Apparatus).

Isobaric-labelled peptides from total proteome and phosphoproteome were fractionated into eight fractions using the Pierce High pH Reversed-Phase Peptide Fractionation Kit (ThermoFisher Scientific) following the manufacturer’s instructions.

#### nLC-MS/MS analyses

Each fraction was analyzed by nanoliquid chromatography coupled to tandem mass spectrometry (nLC-MS/MS, Ultimate 3000 RSLCnano and Q-Exactive HF, Thermo Fisher Scientific) using a 240 min gradient. For this purpose, the peptides were sampled on a precolumn (300 μm × 5 mm PepMap C18, Thermo Scientific) and separated in a 200 cm µPAC column (PharmaFluidics). The MS and MS/MS data were acquired by Xcalibur version 2.9 (Thermo Fisher Scientific).

#### Data analysis

Phosphopeptide enrichment was performed twice, generating two technical replicates, which allowed the identification of a higher number of unique phosphopeptides [11]. A very good reproducibility was observed between the two technical replicates (Pearson's *r* = 0.65; Figures S1A and S1B), allowing their combination for downstream statistical analysis.

Peptides and proteins were identified and quantified using MaxQuant version 1.6.17.0 [12], the UniProt database (*Homo sapiens* taxonomy, 20211014 download) and the database of frequently observed contaminants embedded in MaxQuant. Trypsin/P was chosen as the enzyme and 2 missed cleavages were allowed. Peptide modifications allowed during the search were: Carbamidomethyl (C, fixed), Acetyl (Protein N-term, variable), Oxidation (M, variable), and Phospho (STY, variable). Minimum peptide length and minimum number of razor + unique peptides were respectively set to seven amino acids (AA) and one. Maximum false discovery rates—calculated by employing a reverse database strategy—were set to 0.01 at peptide-spectrum match, protein and site levels. Peptides and proteins identified in the reverse and contaminant databases, and proteins only identified by site were discarded. Only class I phosphosites (localization probability ≥ 0.75) and phosphosites and proteins quantified in all replicates of at least one condition were further processed. After log2 transformation, extracted corrected reporter abundance values were normalized by Variance Stabilizing Normalization (vsn) method in Prostar [13].

### Phosphoproteomic data quality control (QC)

Output of Prostar was loaded into R software (version 4.2.1) (https://www.R-project.org/). Initial quality control was performed using Principal component analysis (PCA), unsupervised clustering (hierarchical clustering and k-means clustering), and sample cross-correlation. Function ‘removeBatchEffects’ of ‘limma’ package (version 3.54.2) [14] was used to correct for batch effects between treatments that were performed on two different days, and batch effects were denoted using design matrix in the downstream analysis. As sample BMP9-3 came as possible outlier during QC (Fig. S1C), ‘lof’ function of ‘dbscan’ package (version 1.1–11) [15] was used to measure local outlier factor (LOF). In addition to LOF, distance from the median in PCA space was used to determine that BMP9-3 is indeed the outlier. Same process was repeated for both technical replicates and for the proteomic data. QC was repeated to confirm that batch effects and outliers were successfully treated. PCA of all phosphosite abundances clearly distinguished non-stimulated (NS) samples from those stimulated with BMP9 or BMP10, but could not distinguish BMP9 from BMP10 stimulated samples (Figure S1D). Analysis of the phosphorylated amino acid distribution showed that 88% of the identified phosphosites were on serine (Ser), 10.2% on threonine (Thr), and 1.8% on tyrosine (Tyr) (Figure S1E), consistent with the expected abundance of these phosphorylated residues in eukaryotic cells [16]. The majority of these phosphosites were singly phosphorylated (67% singly, 24.8% doubly, and 8.2% triply) (Figure S1E).

### Differential phosphorylation analysis (DPA)

DPA was performed using ‘limma’ package [14]. Phosphoproteomic data consisted of two technical replicates, with 7,565 phosphosites quantified in both technical replicates (“overlapping phosphosites” in further text) in addition to 2,326 phosphosites that were quantified only from technical replicate 1 and 2,888 from technical replicate 2. DPA was performed on each technical replicate individually, as well as on overlapping phosphosites (“combined analysis” in further text). Design matrix was used to denote batch effects, and in case of overlapping phosphosites, technical replicates. All P.values were corrected for multiple testing using Benjamini–Hochberg procedure, to obtain adjusted P.values (FDR). Log fold change values and P-adjusted values to be used in downstream analyses were selected such that: 1) For overlapping phosphosites, values were retrieved from the combined analysis; 2) For other phosphosites, values were retrieved from the analysis corresponding to dataset in which phosphosite was quantified.

Phosphosite is considered differentially expressed if FDR value was lower than 0.05, and absolute log fold change value was higher than or equal to 0.1. Threshold of 0.1 is selected such that 90% of the phosphosites, which have an FDR value lower than 0.05, fall above this threshold.

For the proteomic data, differential expression analysis was performed using “limma” package [14]. Since batch effects were detected in proteomic data as well, design matrix was used to denote batch effects.

### Gene Ontology (GO) and WikiPathway over-representation analyses

GO over-representation analysis was performed using list of genes that encode for proteins which were differentially phosphorylated. Analysis was performed using the Metascape online tool (https://metascape.org/) [17], accessed on 24th of August, 2023, using GO Biological Processes (BP) or WikiPathways. Enrichment analysis was performed with the following settings: minimum overlap of 5 genes, P-value cut-off of 0.05, minimum enrichment of 1.5, while using all genes as the background. Using the online tool incorporated in Metascape, significantly enriched GO-BPs were clustered based on semantic similarity. Additionally, clustering was further manually fine-tuned and annotated to improve understandability.

### KinSwing analysis

KinSwing analysis was performed using the ‘KinSwingR’ package (version 1.16.0) [18]. As an input, KinSwingR takes results of DPA (Fold change and P.value) as well as Position Weight Matrices (PWM). PWM was built with the “buildPWM’ function based on kinase-substrate interaction data from the PhosphoSitePlus database included in KinSwingR package. Redundancies in data, caused by multiplicity, were removed by retaining ones that have higher Fold change and higher –log_10_ (*p*.value). KinSwing analysis was performed on flanking sequence (SITE_ ± 7_AA).

### Post Translational Modification Signature Enrichment analysis (PTM-SEA)

Gene set enrichment analysis (GSEA) was performed using the ClusterProfiler (version 4.6.2) [19] R package. PTMsigDB [20] was obtained from WebGestalt using the “WebGestaltR” (version 0.4.6, accessed on 24/08/2023) R package. GSEA sorting vector was built using the flanking sequence (SITE_ ± 7_AA), and sorted based on log fold change obtained from DPA. Redundancies due to multiplicity were dealt with in same way as for KinSwingR analysis (see above). GSEA exponent (weights) parameters was set to 0.75 (1 default) in order to flatten the log fold change values distribution.

### Site-directed mutagenesis

Plasmids encoding ALK1 mutations were generated using PCR-based site-directed mutagenesis, whereby a pcDNA3-1( +) plasmid encoding the wild-type (WT) N-terminal HA-tagged ALK1 was modified using the QuikChange Lightning kit (Agilent) following the manufacturer’s instructions. The mutated plasmids were subsequently subjected to full sequencing (Eurofins) to verify the presence and location of the mutations. Primer sequences utilized for mutagenesis are listed in supplementary Table S1.

### BRE luciferase assay

NIH-3T3 cells were transfected in Opti-MEM (Invitrogen) using lipofectamine 2000 (Invitrogen) with 75 ng pGL3(BRE)2-luc, 30 ng pRL-TKluc and 5 ng of plasmids encoding either HA-tagged WT or generated mutants for ALK1. Five hours post transfection, cells were stimulated with or without recombinant human BMP10 (100 pg/mL) for 18 h. Firefly and renilla luciferase activities were sequentially measured with Twinlite Firefly and Renilla Luciferase Reporter Gene Assay System (Perkin Elmer) using the TECAN SPARK multimode microplate reader (Thermofisher). Final luciferase activities were reported as folds of firefly luciferase activities normalized to renilla luciferase activities.

### Chemicals

Chemical inhibitors used were commercially available and listed in the key resources Table S2. SB203580, PF3644022, and DRB were dissolved in DMSO. Actinomycin D was dissolved in ethanol. LDN193189 was dissolved in water. The final concentration of DMSO or ethanol in the medium was a maximum 0.5%, and DMSO or ethanol were used as vehicle controls at the same final concentration.

### Western blotting

Following stimulations with BMP9 or BMP10, cells were washed twice with ice-cold phosphate-buffered saline (PBS) and lysed in a Tris-based lysis buffer supplemented with protease inhibitor cocktail and phosphatase inhibitor cocktails 2 and 3. Equal protein quantities (10-20 µg) were then resolved by SDS–polyacrylamide gel electrophoresis (SDS-PAGE) on 4–20% Precast Protein Gels (Bio-rad) and transferred onto a nitrocellulose membrane using Mini Trans-Blot system (Bio-rad). Membranes were blocked with instant block buffer (Sigma) and subsequently probed with indicated primary antibodies (key resources Table S2). Membranes were incubated with enhanced chemiluminescence substrates (ECL; Bio-rad) or SuperSignal™ West Femto Maximum Sensitivity Substrate (Thermo Scientific™) and images were developed using ChemiDoc™ Imaging System (Bio-Rad). Chemiluminescent signal intensity was quantified with the Image lab software V6.1 (Bio-Rad). All measurements were performed within the linear range and were normalized to the non-phosphorylated total protein or HSP90 loading control.

### RNA extraction and RT-qPCR

Total RNA was extracted using the NucleoSpin RNA kit (Macherey–Nagel) following the manufacturer's instructions. 500 ng of RNA was then reverse-transcribed into cDNA using the iScript cDNA Synthesis Kit (Bio-Rad). Reverse transcription quantitative PCR (RT-qPCR) was then performed using SsoAdvanced Universal SYBR Green Supermix (Bio-Rad) in a CFX96 Real-Time System (Bio-Rad). Data analysis was conducted utilizing the CFX Manager Software V3.1 (Bio-rad), then gene expression levels were calculated utilizing Livak's 2^−ΔΔCt^ method using *HPRT* as housekeeping gene, and results were presented as fold change relative to control unstimulated samples. All primer sequences used for RT-qPCR were pre-designed using primer blast tool and purchased from sigma. Table S1 summarizes all the genes assessed by RT-qPCR with their respective primer mixes.

### RNA interference

HUVECs were transfected with either a scrambled Silencer Negative Control #1 siRNA (siScr or siCTL; AM4611, Ambion), pre-designed siRNA (Thermofisher) directed against human SMAD4 (siSMAD4; assay ID s8405) or human GADD45B (siGADD45B; assay ID s9141) at a final concentration of 10 nM in 1 mL of Opti-MEM (Gibco) using 2.5µL/well Lipofectamine RNAiMAX Transfection Reagent (Invitrogen). 48 h post-transfection, cells were serum starved in EBM2 for 6 h and were then stimulated or not with 10 ng/mL BMP10 for 30 min.

### Cell cycle analysis by flow cytometry

HUVECs were synchronized by a 48 h starvation in EBM2 containing 0.5% FBS. Next, cells were stimulated or not with BMP10 (10 ng/mL) overnight in EBM2 supplemented with 0.5% or 5% FBS. For the analysis of cell cycle progression, actively proliferating cells were detected using Click-iT™ Plus EdU Alexa Fluor™ 647 (Invitrogen) according to the manufacturer’s instructions with minor modifications. Thirty thousand cells were analyzed on the FACS Calibur (BD Biosciences), and data were analyzed using FCS Express.

### Statistical analysis

Graphs and statistical analyses were generated using GraphPad Prism software v8.2.1. All values were represented as the mean ± SEM. Significance was calculated as indicated in the figure legends: ∗ , # *p* < 0.05, ∗ ∗ , ## *p* < 0.01, ∗ ∗ ∗ , ### *p* < 0.001, ∗ ∗ ∗ ∗ , #### *p* < 0.001. At least two independent experiments were performed for each analysis. Statistical tests used and number of independent experiments carried out are indicated in the figure legends.

## Results

### Phosphoproteome profiling of endothelial cells (ECs) stimulated by BMP9 and BMP10

**Fig. 1:**
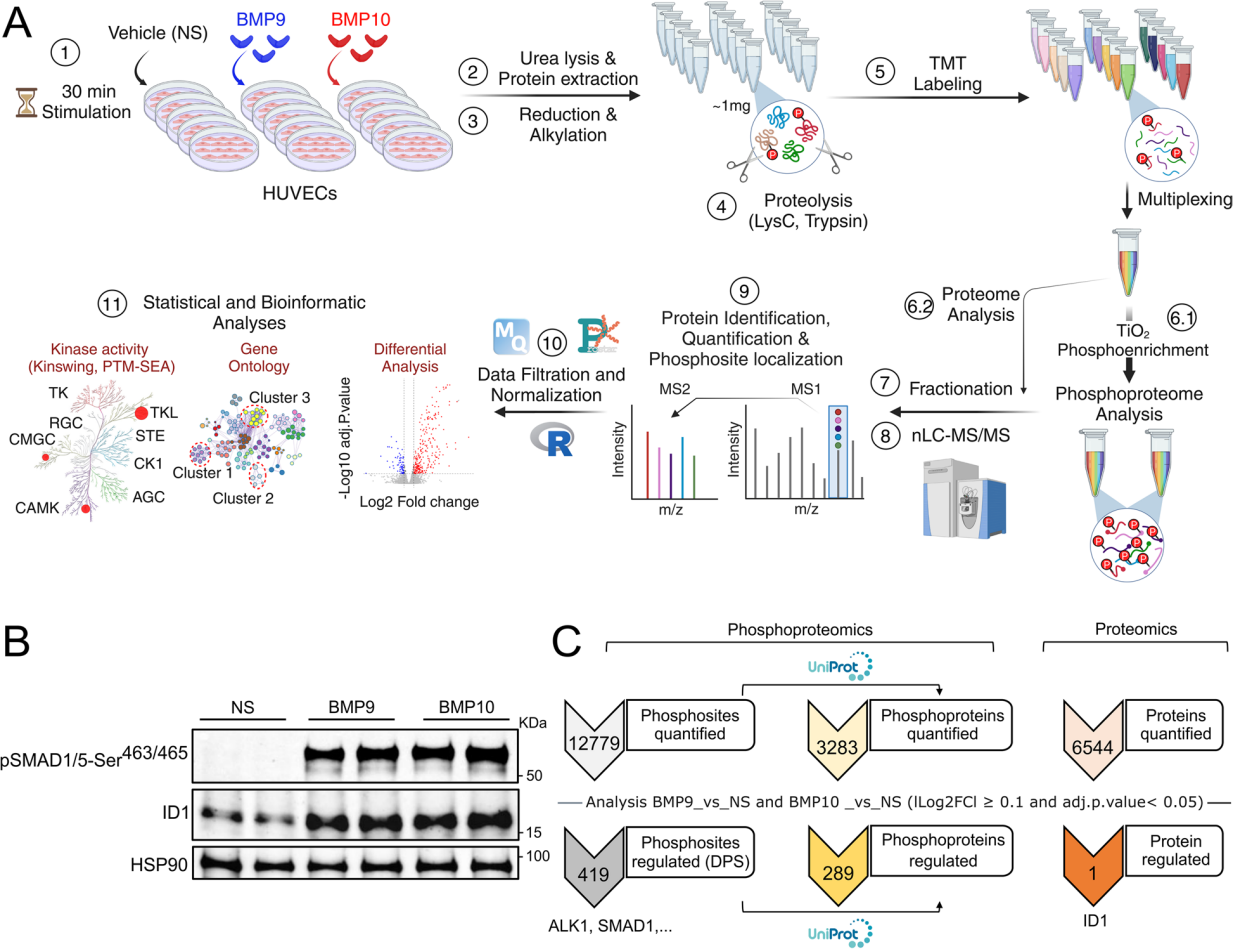
Phosphoproteome profiling of BMP9 and BMP10 stimulation in human umbilical vein endothelial cells (HUVECs). **A** Graphical representation of the experimental workflow for phosphoproteomic analysis. (1) HUVECs were stimulated or not (NS) with 10 ng/mL of BMP9 or BMP10 for 30 min. (2) Lysates from five biological replicates per condition were prepared and (3) subjected to reduction and alkylation, followed by (4) digestion using a combination of two endoproteinases, LysC and trypsin. (5) The resulting peptides were labeled with tandem mass tag (TMT) reagents and pooled for subsequent analysis. Phosphorylated peptides were then enriched using titanium dioxide (TiO2) beads (6.1), while a small portion of the pooled samples was reserved for proteomic analysis (6.2). (7, 8) The proteome and phosphoproteome samples were fractionated and analyzed using liquid chromatography tandem Mass spectrometry (LC–MS/MS). Phosphoproteomic analysis was performed twice on two separate fractions generating two technical replicates. (9–11) Data analysis was then performed using different bioinformatics tools. **B** Western blotting analysis of two biological replicates for each condition (NS, BMP9 or BMP10) used for phosphoproteomic analyses showing the levels of SMAD1/5 phosphorylation and ID1 expression. **C** Upper numbers represent count of identified and quantified phosphosites and their corresponding phosphoproteins annotated in UniProtKB database, as well as that of proteins (proteomic analysis) across all samples. Bottom numbers represent total count of differentially phosphorylated sites (DPS) and proteins by both BMP9 and BMP10

To analyze in a comprehensive manner early phosphorylation events regulated by BMP9 and BMP10, we stimulated human umbilical vein endothelial cells (HUVECs) with 10 ng/mL of BMP9 or BMP10 for 30 minutes (min). We selected a relatively high dose of BMP (10 ng/mL), which falls within the range of circulating active BMP9 and BMP10 [21], and a 30-min stimulation period, which corresponds to the plateau of SMAD1/5 phosphorylation. Our experimental design consisted of five biological replicates under three conditions: non-stimulated (NS), BMP9-stimulated, or BMP10-stimulated cells (Fig. 1A). Effective stimulations of HUVECs by BMPs were validated by western blot analysis, confirming SMAD1/5 phosphorylation and expression of ID1, a well-known downstream target of BMP9 and BMP10 signaling [22] (Fig. 1B). Deep phosphoproteomic analysis of these samples allowed to identify and quantify 12,779 phosphopeptides containing class I phosphosites (localization probability > 75%), mapped to 3,283 proteins in the UniprotKB database (Fig. 1C, Table S3). Among the detected phosphosites, 550 were not previously documented in the PhosphoSitePlus database [23] (*Homo sapiens* taxonomy). Differential phosphorylation analysis highlighted 419 differentially phosphorylated sites (DPSs, **|**Log2FC Stimulated_*vs*_Non Stimulated**|**≥ 0.1 and adj.p-value < 0.05) in cells stimulated by BMP9 or BMP10 compared to NS cells, corresponding to 289 different proteins (Fig. 1C, Table S3).

Complementary MS-based quantitative analysis of total proteome of the same samples identified and quantified 6,544 proteins (Fig. 1C, Table S3), among which, only the transcription factor ID1 was found to be differentially regulated by BMP9 and BMP10 compared to NS cells (Fig. 1C). Thus, none of the differentially regulated phosphosites were located on proteins showing changes in their abundance upon stimulation of HUVECs with BMP9 or BMP10, supporting that the observed alterations in protein phosphorylation were not influenced by changes in the expression levels of the respective proteins (Table S3).

**Fig. 2:**
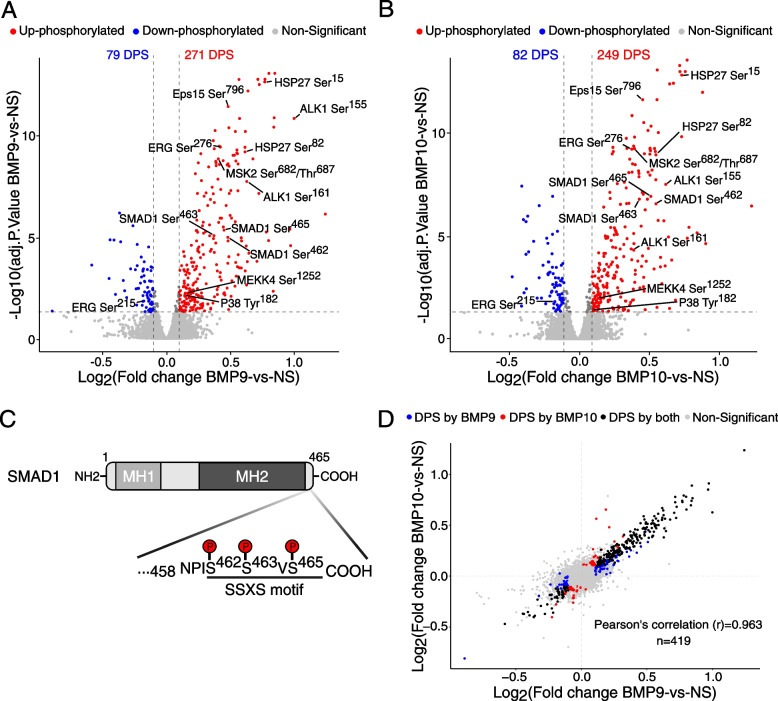
Analysis of phosphoproteomic changes in response to BMP9 and BMP10 in HUVECs. **A** and **B** Volcano plots representing the log2 fold change in the abundance of phosphopeptides plotted against the –log10 adj. P.value, showing differentially phosphorylated sites (DPS) which are down-phosphorylated (blue) or up-phosphorylated (red) in response to BMP9 (**A**) or BMP10 (**B**) stimulation. DPSs from the canonical ALK1/SMAD1 signaling pathway as well as those which will be further studied in the work are annotated. **C** SMAD1 linear structure showing the MH1 (MAD homology domain 1) and MH2 domain, along with the C-terminal SSXS motif sites highlighted in panels **A** and **B**. **D** Scatter plot comparing log2 fold change values of DPS regulated by BMP9 and BMP10. Pearson’s correlation (r) is reported

Differential phosphorylation analysis identified 271 and 249 up-regulated phosphosites, versus 79 and 82 down-regulated phosphosites, in response to BMP9 and BMP10, respectively (Fig. 2A and B). Among these, we identified phosphorylation of the activating residues on the C-terminus of SMAD1 (Ser^462/463/465^), a direct substrate of ALK1 [24], thus validating our phosphoproteomic approach (Fig. 2A-C). Comparison of phosphosite abundances in BMP9- or BMP10-stimulated cells *versus* NS cells indicated that BMP9 and BMP10 stimulations induced similar cell response at the phosphoproteome level, as evidenced by the high Pearson’s correlation coefficient (r) = 0.963 (Fig. 2D). Interestingly, we identified up-regulated DPSs, as expected from the BMP9/BMP10-induced activation of the ALK1 kinase, but also down-regulated DPSs (Figs. 2A and B). Accordingly, our analysis revealed differential phosphorylation of two phosphatases, ILKAP-Ser^28^ (Integrin-Linked Kinase-Associated Phosphatase) and CTDSPL2-Ser^13^ (potential phosphatase), in addition to fourteen differentially phosphorylated kinases (Table S4), six of which containing functionally characterized phosphosites (P38α-Tyr^182^, ATR-Ser^435^, MSK2-Thr^687^, P70S6K-Ser^447^, LIMK-Ser^310^ and SLK-Ser^189^). As expected, among these kinases, this study identified sites within the type I receptor ALK1 (Fig. 2A and B). However, these sites (Ser^155/161^) lie within the juxtamembrane region of ALK1, which does not correspond to the known phosphorylation sites within the GS domain of type I receptors (Figure S2A) [25]. Given that BMP9 and BMP10 induced similar phosphoproteomic changes (Fig. 2D), all further experiments were conducted in response to BMP10 stimulation only, except when indicated.

To assess the functional significance of these two identified ALK1 phosphosites (Ser^155/161^), we generated mutants that can no longer be phosphorylated at these sites by replacing serine with alanine residues (S155A, S161A, and S155A-161A), or by introducing a negative charge mimicking their constitutive phosphorylation by substituting serine to aspartic acid (S155D, S161D, and S155D-161D). As a positive control, we included the constitutively active mutation ALK1-Q201D [26]. Using the ID1 promoter-derived BMP response element (BRE)-luciferase assay [27], we found that these mutants exhibited a response similar to that of wild type (WT) ALK1 (Figures S2B and S2C), showing that these two ALK1 phosphosites (Ser^155/161^) are not implicated in the canonical SMAD1/5 pathway in ECs.

### Bioinformatics-based biological interpretation of BMP9/10-induced phosphoproteome changes in ECs

**Fig. 3:**
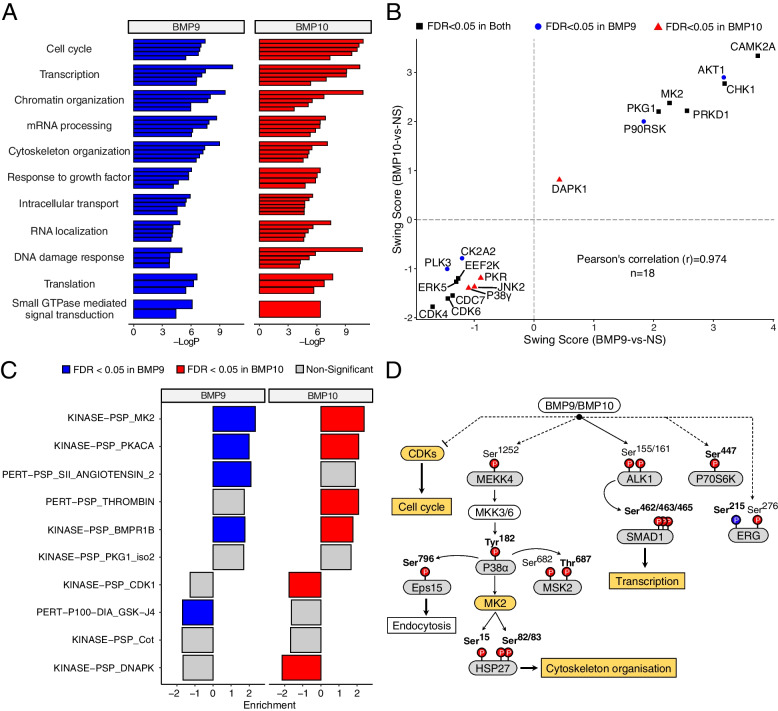
Bioinformatic analyses of the phosphoproteomic changes in response to BMP9 and BMP10 in HUVECs. **A** Gene ontology on biological processes (≥ 1.5-fold enrichment, *P* ≤ 0.05) was conducted using Metascape, based on DPS gene list for BMP9 and BMP10. Only the common clusters between BMP9 and BMP10 are shown, with the top 5 terms for each cluster. The full list of clusters and their corresponding terms are outlined in Table S5. **B** Scatter Plot of KinSwing scores of kinases whose activities were predicted to be significantly changed in response to BMP9 and/or BMP10. Positive swing scores indicate predicted activation of a kinases, while negative scores indicate predicted under-activation of a kinase. **C** Post translational modification signature enrichment analysis (PTM-SEA) performed using PTMsigDB. Bar plot represents the top signatures enriched in BMP9 and/or BMP10 stimulated HUVECs compared to NS cells, ordered in terms of average FDR from both comparisons. Each bar represents a signature (kinase or perturbation). PERT, Perturbation; PSP, Phosphositeplus. **D** Hypothetical signaling framework in response to BMP9 and BMP10 based on phosphoproteomic analysis and literature-based data. Phosphorylated residues with increased abundance in response to BMP9 and BMP10 are marked in red, while those displaying decreased abundance are marked in blue. Bold numbers represents phosphosites with low-throughput papers derived from PhosphositePlus. Grey curved rectangles represent BMP9 and BMP10-derived differentially phosphorylated proteins, white ones are from literature. Orange curved rectangles: bioinformatic-predicted kinases (KinSwing or PTM-SEA). Solid orange rectangles: GO-BP analysis-identified processes, white from literature. Dashed lines signify uncharacterized mechanisms

To understand the underlying biological processes (BP) regulated by BMP9- and BMP10-mediated phosphoproteome modifications in ECs, we performed gene ontology enrichment analysis (GO-BP) with the 289 differentially phosphorylated proteins. Functional annotation clustering of the enriched terms produced 13 clusters for BMP9 and 14 for BMP10, with 11 clusters shared between both ligands (Fig. 3A and Table S5). The first cluster contained several terms related to cell cycle regulation. Moreover, we identified several clusters with terms related to transcription regulation, chromatin organization, and mRNA processing, supporting an important impact of BMP9 and BMP10 stimulation on the regulation gene expression in ECs (Fig. 3A and Table S5).

To identify the potential upstream kinases responsible for the significant changes observed in the phosphoproteome of ECs upon BMP9 and BMP10 stimulation, we used the KinSwing approach, which integrates sequence windows of phosphosites with kinase-substrate motifs [18]. This analysis identified 10 kinases whose activity is predicted to be modified in ECs in response to both BMP9 and BMP10 stimulations (Fig. 3B and Table S5). In accordance with our GO-BP analysis that highlighted the cell cycle cluster (Table S5), KinSwing analysis revealed a significant under-activation of CDK4/6 and CDC7, key regulatory kinases involved in the G1/S transition of the cell cycle [28, 29] (Fig. 3B, Table S5). Moreover, protein kinase R (encoded by *EIF2AK2*), which is involved in the inhibition of protein synthesis [30], and ERK5 (encoded by *MAPK7*), belonging to the MAPK family, were also predicted to be deactivated upon BMP9/10 stimulation (Fig. 3B, Table S5). On the other hand, CAMK2A (calcium/calmodulin-dependent protein kinase II alpha), CHK1 (checkpoint kinase 1), MAPKAPK2/MK2 (a downstream kinase of P38/MAPK pathway), PRKD1 (Protein Kinase D1), and PKG1 (cGMP-dependent protein kinase) were predicted to be positively regulated. PRKD1 plays a role in DAG/PKC signaling [31], while PKG1 is involved in eNOS/cGMP signaling and has been linked to aortic aneurism and arterial hypertension [32].

Next, we performed a post-translational modification signature enrichment analysis (PTM-SEA) using PTMsigDB to further define the associations between the regulation of each phosphosite and molecular signatures of perturbations, kinase activities, and pathways obtained from curated published datasets [20]. As expected, this analysis predicted that, in ECs, BMP9/10 stimulation activates BMP type I receptor kinase (BMPRIB) due to the shared SMAD1 phosphorylation sites between different BMP type I receptors. Additionally, this analysis highlighted two top kinases, MK2 (already identified using the KinSwing approach) and PKA (PKACA) (Fig. 3C, Table S5).

Taking into account different targets identified in the phosphoproteomic analysis and the biological interpretations generated by our bioinformatic analyses, we could propose a hypothetical model of the BMP9/10-mediated signaling framework in ECs (Fig. 3D). In addition to the activation of the canonical SMAD1 pathway, our analysis highlighted the involvement of the MEKK4/P38α/MK2 signaling pathway, with at least three P38 downstream targets among the top DPSs (Fig. 2A and B): the kinase MSK2, HSP27, a member of the small heat shock protein family acting as a chaperone for correct protein folding and playing an important role in cytoskeleton organization [33], and Eps15 (epidermal growth factor receptor pathway substrate 15), which has been shown to be involved in the endocytosis of cell surface receptors, including EGFR [34]. In addition to the predicted under-activation of CDKs (Fig. 3B and C), we also observed the differential phosphorylation of P70S6K (Ser^447^) (Table S4), which belongs to the mTOR pathway. Notably, its downstream target RPS6 has been previously identified to be differentially phosphorylated in HHT patients [35]. Furthermore, we identified differential phosphorylation of the endothelial transcription factor ERG (Ser^215^ and Ser^276^) (Fig. 3D).

### BMP10 induces distinct phosphorylation changes in ERG

**Fig. 4:**
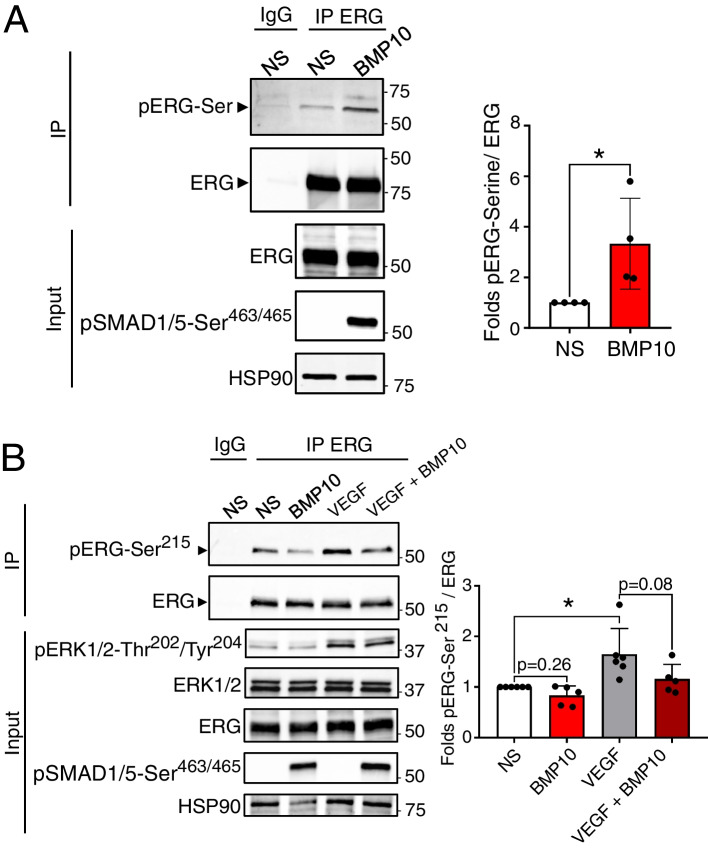
ERG is differentially phosphorylated by BMP10 in HUVECs. **A** HUVECs were stimulated with 10 ng/mL BMP10 or not (NS) for 30 min. Cell extracts were subjected to immunoprecipitation (IP) using ERG antibody, followed by WB using pan phosphoserine (pSer) and ERG antibodies. Total serine phosphorylation of ERG was quantified and normalized to total ERG levels. IgG represents lysates subjected to IP using isotype control antibody. Whole cell extracts (input) were subjected to WB analysis using antibodies against pSMAD1/5, ERG and HSP90. Data are presented as mean folds (BMP10-vs-NS) ± SEM of *n* = 4 independent experiments.*P < 0.05 using Kolmogorov–Smirnov test. **B** HUVECs were stimulated with 10 ng/mL BMP10, or VEGF (40 ng/mL), BMP10 + VEGF (10 and 40 ng/mL, respectively) or not (NS) for 30 min. Cell extracts were subjected to IP using ERG antibody, followed by WB using antibodies against ERG-Ser215 and ERG. Quantification of ERG phosphorylation reflects the normalized signal for the pERG-Ser215 to total ERG from each sample. IgG represents lysates subjected to IP using isotype control antibody. Whole cell extracts (input) were subjected to WB analysis using antibodies against pERK1/2-Thr202/Tyr204, ERK1/2, ERG, pSMAD1/5, and HSP90 (loading control). Data are presented as mean folds (BMP10, VEGF or VEGF + BMP10-vs-NS) ± SEM of at least 5 independent experiments. Statistical analyses were performed using two-way ANOVA followed by Sidak's multiple comparisons post-test. *P < 0.05

Recently, ERG has emerged as a key transcriptional factor of endothelial function, playing vital roles in angiogenesis, vascular stability, as well as the differentiation and maintenance of the endothelial lineage [36]. In this work, we identified differential phosphorylation of ERG at two different sites which, unexpectedly, exhibited opposite patterns. Particularly, Ser^215^ was found to be less phosphorylated upon stimulation of ECs with BMP9 and BMP10, whereas Ser^276^ was found to be more phosphorylated (Fig. 2A and B). Unfortunately, no specific anti-phospho-ERG-Ser^276^ antibody is available. Nevertheless, BMP10-induced ERG phosphorylation was validated by western-blotting of total serine phosphorylation after ERG immunoprecipitation (Fig. 4A). On the other hand, using an anti-phospho-ERG-Ser^215^ antibody generously provided by Dr. P. Hollenhorst [37], we were able to confirm that BMP10 stimulation down-regulates the phosphorylation of ERG at Ser^215^ (Fig. 4B). Additionally, we validated the previously reported VEGF-induced phosphorylation [38] of ERG-Ser^215^ and found that the addition of BMP10 led to a reduction in this VEGF-induced phosphorylation (Fig. 4B).

### BMP10 signaling in HUVECs drives the activation of the P38-MK2 axis

**Fig. 5:**
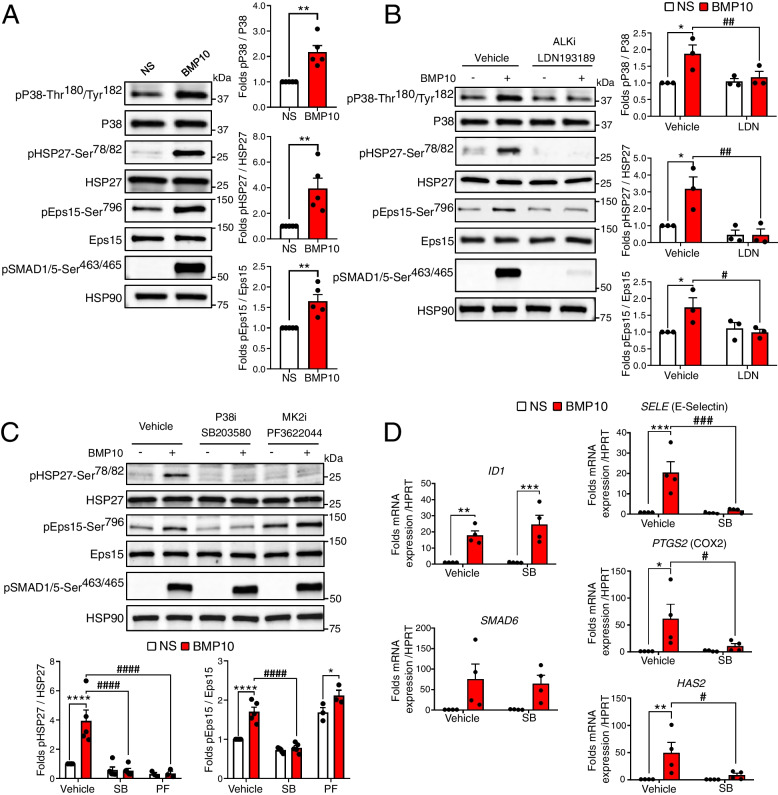
BMP10 signaling in HUVECs drives the activation of the P38-MK2 axis. **A** HUVECs were stimulated with 10 ng/mL BMP10 or not (NS) for 30 min. Cell extracts were subjected to western blotting (WB) analysis using antibodies against phosphorylated (p) P38-Thr180/Tyr182, P38, pHSP27-Ser78/82, HSP27, pEps15-Ser796, Eps15, pSMAD1/5-Ser463/465 and HSP90 (loading control). Quantification of phosphorylation for P38, HSP27 and Eps15 reflects the normalized signal for the phosphorylated protein to total protein content, presented as mean fold change (BMP10-vs-NS) ± SEM of *n* = 5 independent experiments. **P < 0.01 using Kolmogorov–Smirnov test. **B** and **C** HUVECs were pretreated with selective ALK1/2/3/6 inhibitor LDN193189 (LDN, 5 µM) (Panel **B**), P38 inhibitor SB203580 (SB, 10 µM) or MK2 inhibitor PF3622044 (PF, 5 µM) (Panel **C**), or left untreated (vehicle) for 30 min. Subsequently, cells were stimulated with 10 ng/mL BMP10 or not (NS) for an additional 30 min. Cell lysates were then analyzed by WB using the indicated antibodies and quantification was performed as explained in panel **A**. Data shown represent mean folds ± SEM of at least 3 independent experiments. **D** HUVECs were pretreated with P38 inhibitor SB203580 (SB, 10 μM) or left untreated (vehicle) for 30 min. Cells were then stimulated with 10 ng/mL BMP10 or not (NS) for 4 h. Real-time quantitative polymerase chain reaction analysis was then performed to determine mRNA expression of ID1, SMAD6, SELE (E-Selectin), PTGS2 (COX2), and HAS2. Target gene expression was normalized to HPRT using 2-ΔΔCt method and presented as fold induction (BMP10-vs-vehicle) ± SEM of *n* = 4 independent experiments. Statistical analyses for panels **B**, **C** and **D** were performed using two-way ANOVA followed by Sidak's multiple comparisons post-test. For all panels: *,#*P* < 0.05; **,##*P* < 0.01; ***,###*P* < 0.001; ****,####*P* < 0.0001. *: BMP10-vs-NS; #: vehicle-vs-inhibitor (LDN, SB or PF)

Our phosphoproteomic screen identified the differential phosphorylation of three kinases belonging to the MAPK pathway (MEKK4, P38α and MSK2) and of two potential substrates of this pathway (HSP27 and Eps15, Fig. 2A and B), suggesting that BMP9 and BMP10 could signal through this pathway in ECs. By Western blot analysis, we successfully confirmed the phosphorylation of P38-Thr^180^/Tyr^182^ and HSP27-Ser^78/82^ in HUVECs stimulated for 30 min with BMP10 (Fig. 5A). We also validated Eps15-Ser^796^ phosphorylation using a specific phospho-antibody kindly provided by Dr Sakurai [39] (Fig. 5A). These phosphorylation events were also validated in response to BMP9 stimulation (Figure S3). We next tested different concentrations of BMP10 and observed a similar dose–response for phosphorylation of SMAD1/5, P38, HSP27 and Eps15, which started to be detectable from 0.1 ng/mL and reached a plateau at 1 ng/mL (Figure S4).

To assess the involvement of ALK1 in this signaling, we treated cells with LDN193189, an inhibitor known to affect ALK1 kinase activity along with ALK2, 3, and 6 [40]. Importantly, we found that the BMP10-induced increase in phosphorylation at specific sites of P38, HSP27 and Eps15 was abrogated by LDN193189 treatment (Fig. 5B). As HSP27 has been shown to be phosphorylated by MK2 through the P38/MK2 signaling axis [41], one of the top enriched kinases revealed by our bioinformatic analysis (Fig. 3B and C), and since Eps15-Ser^796^ has been shown to be phosphorylated in response to TNF-α via the P38 MAPK pathway [39], we tested whether the specific phosphorylation of these proteins upon BMP9/10 stimulation of ECs was dependent on P38 and MK2 kinases using specific inhibitors. As expected, SMAD1/5 phosphorylation was not affected neither by the P38 inhibitor (SB203580) nor the MK2 inhibitor (PF3622204) (Fig. 5C). On the other hand, we found that HSP27 phosphorylation was inhibited by both inhibitors, while Eps15 phosphorylation was only inhibited by the P38 inhibitor (Fig. 5C).

Notably, P38 (*MAPK14*) was identified in several enriched GO-BP categories associated with transcriptional regulation revealed by our bioinformatic analyses (Table S5). We then tested whether it may play a role in BMP10-induced gene regulation. To this end, HUVECs were stimulated by BMP10 for 4 h with or without pretreatment with the P38 inhibitor SB203580. We found that inhibition of P38 did not affect *ID1* nor *SMAD6* mRNAs expression, two canonical targets of BMP9 and BMP10 in ECs (Fig. 5D). On the other hand, the mRNA expression of *SELE* (E-selectin), *PTGS2* (COX2, cyclooxygenase 2), and *HAS2* (hyaluronan synthase 2) were found to be dependent on P38 activity (Fig. 5D). Collectively, these results reveal that BMP10 activates P38 MAPK signaling, resulting in the phosphorylation of HSP27-Ser^78/82^ by P38/MK2 and Eps15-Ser^796^ by P38. Moreover, P38 activation mediates the regulation of expression of a subset of BMP10 target genes.

### BMP10 signaling in HUVECs induces activation of the P38 MAPK pathway via GADD45β expression

**Fig. 6:**
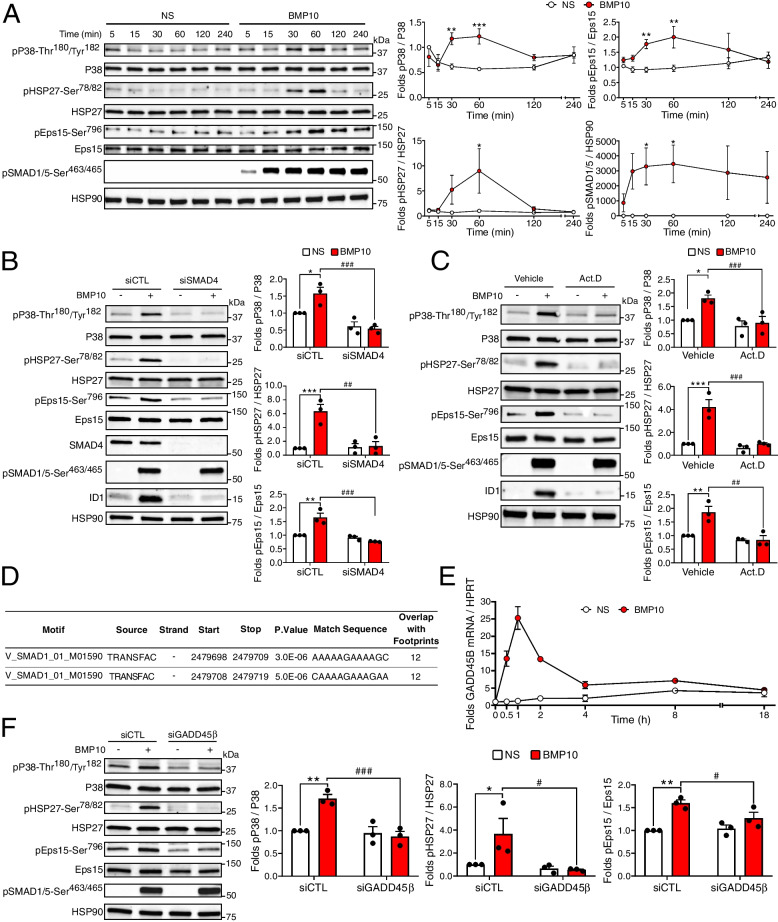
BMP10 signaling in HUVECs induces a delayed activation of the P38 MAPK pathway via GADD45β expression. **A** Cells were stimulated with 10 ng/mL BMP10 or not (NS). Cell extracts were subjected to WB analysis using antibodies against pP38-Thr180/Tyr182, P38, pHSP27-Ser78/82, HSP27, pEps15-Ser796, Eps15, pSMAD1/5-Ser463/465 and HSP90. Data are presented as fold change of each sample ± SEM relative to NS 5 min, *n* ≥ 3. **B** HUVECs were treated either with scrambled siRNA (siCTL) or siSMAD4 and then stimulated with BMP10 or not for 30 min. Cell extracts were analyzed by WB was performed as in panel **A**. **C** HUVECs were pre-treated either with vehicle or the transcription inhibitor actinomycin D (Act.D, 5 μM) for 30 min, then stimulated with BMP10 or not for another 30 min. Cell extracts were analyzed by WB as in panel **A**. Data are presented as mean folds ± SEM of *n* = 3. **D** SMAD1 binding sites and number of overlaps with footprints within GADD45β promoter extracted from transcription factor target gene database (TFBS). **E** HUVECs were stimulated with BMP10 or not (NS) and GADD45β mRNA expression was then assessed by RT-qPCR. The level of GADD45β mRNA expression was normalized to HPRT and represented as fold induction of each NS and BMP10 stimulated sample relative NS 0 min ± SEM of *n* = 2. (**F**) HUVECs were treated with either scrambled siRNA (siCTL) or siGADD45β and then stimulated with BMP10 or not (NS) for 30 min. Cell extracts were analyzed by WB as in panel **A**. Data are presented as mean folds ± SEM of *n* = 3. Statistical analysis for panel **A** was performed using Kruskal Wallis with Sidak’s post-test. Statistical analyses for panels **B**, **C** and **F** were performed using two-way ANOVA followed by Sidak's multiple comparisons posttest. For all panels: *,#*P* < 0.05; **, ##*P* < 0.01; ***, ###*P* < 0.001. *: BMP10-vs-NS; #: siCTL-vs-siSMAD4 or siGADD45β, or Vehicle-vs-Act.D

To obtain a comprehensive understanding of the molecular mechanisms responsible for phosphorylation of P38, HSP27 and Eps15 in ECs upon BMP10 stimulation, we performed a time-course analysis. As previously shown, we found that pSMAD1/5 was detectable after 5 min of BMP10 stimulation, reaching a plateau from 30 min until 240 min (Fig. 6A). On the other hand, phosphorylation of P38, HSP27 and Eps15 started to be detected 30 min post BMP10 stimulation, peaked at 1 h, and returned to basal levels after 4 h (Fig. 6A).

Considering the delay between SMAD1/5 activation and the phosphorylation of P38, HSP27 and Eps15, we tested whether these phosphorylation events were SMAD-dependent using siRNA directed against SMAD4, the central mediator of SMAD signaling. We confirmed the efficacy of siSMAD4 by Western blotting, showing a strong reduction in SMAD4 expression (Fig. 6B). As expected, SMAD4 depletion did not impact SMAD1/5 phosphorylation triggered by BMP10, but completely abrogated ID1 expression (Fig. 6B). Interestingly, we found that the phosphorylation of P38, HSP27 and Eps15 were also inhibited by SMAD4 silencing (Fig. 6B), showing that these phosphorylation events were dependent on SMAD signaling. We thus tested whether the delay in BMP10-induced phosphorylation of P38, HSP27 and Eps15 required a transcriptional step. To this end, HUVECs were pretreated with the transcription inhibitor actinomycin D 30 min prior to BMP10 addition. As expected, we found that SMAD1/5 phosphorylation induced by BMP10 was not affected by actinomycin D pretreatment, while ID1 expression was dramatically reduced (Fig. 6C). On the other hand, we found that BMP10-induced phosphorylation of P38, HSP27 and Eps15 were all inhibited by actinomycin D pretreatment (Fig. 6C). These results were further confirmed using DRB (5, 6-Dichlorobenzimidazole 1-β-D-ribofuranoside), another transcription inhibitor (Figure S5). Collectively, these data demonstrate that BMP10 triggers the activation of the P38 pathway through a SMAD-dependent transcriptional step in HUVECs, subsequently leading to the phosphorylation of HSP27-Ser^78/82^ by P38/MK2 and Eps15-Ser^796^ by P38.

It has been demonstrated that delayed activation of P38 MAPK through TGF-β stimulation could depend on SMAD-mediated expression of GADD45β (growth arrest and DNA damage inducible beta), which activates MEKK4, an upstream MAPKKK regulating P38 activity [42, 43]. Notably, our phoshoproteomic analysis identified MEKK4 as differentially phosphorylated in response to BMP9 and BMP10 stimulations in HUVECs (Fig. 2A and B). Moreover, in-silico analysis of the promoter region of *GADD45β* using transcription factor (TF)-target gene database [44] revealed the presence of two SMAD1 binding sites (Fig. 6D). To explore further if GADD45β plays a role in BMP10-induced P38 activation in ECs, we assessed whether BMP10 stimulation regulates *GADD45β* mRNA expression using RT-qPCR. We found that BMP10 rapidly induced *GADD45β* mRNA expression within 30 min, peaked at 1 h, and returned back near basal levels after 4 h (Fig. 6E). This positive regulation was detectable at concentrations as low as 0.1 ng/mL of BMP10, reaching a plateau beyond 1 ng/mL (Figure S6A). We next tested the effect of siRNA targeting GADD45β expression (Figure S6B) on BMP10-induced phosphorylation of P38, HSP27 and Eps15. Interestingly, we found that these phosphorylation events were all dependent on GADD45β expression, while pSMAD1/5 was not (Fig. 6F). Together, these results strongly support that BMP10 activates the P38 MAPK pathway via SMAD-dependent expression of GADD45β.

### BMP10 inhibits the G1/S cell cycle transition in HUVECs

**Fig. 7:**
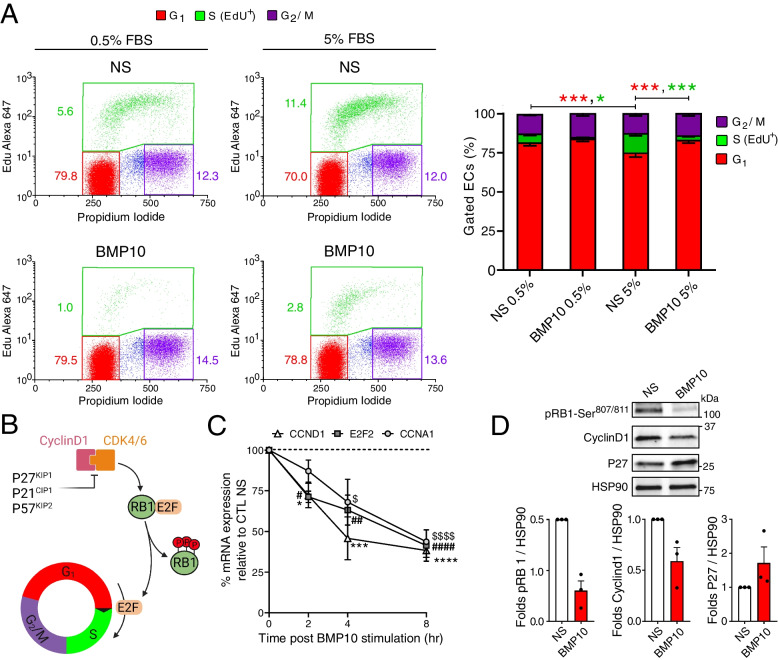
BMP10 inhibits the G1/S cell cycle transition in HUVECs. **A** HUVECs were synchronized at the G1 phase by serum starvation for 48 h. Subsequently, cells were then either replenished with 5% FBS or 0.5% FBS in the presence or absence (NS) of 10 ng/mL BMP10 added overnight. Cells were then treated with EdU for 1.5 h, trypsinized, and labeled for EdU (S phase) and propidium iodide (PI). Left panel: flow cytometry analysis assessing the distribution of different stages of the cell cycle (G1, S, G2/M). Right panel: Quantification of different cell cycle stages. Data are represented as mean % of each stage ± SEM of *n* = 4 and analyzed using two-way ANOVA with Benjamin Hochberg multiple comparisons post-test. Red asterisks represent statistical significance from G1-phase population comparisons between different conditions, while green asterisks represent that for S-phase population comparisons. * *P* < 0.05; *** *P* < 0.001. **B** Schematic representation of different key proteins implicated in the G1/S transition of the cell cycle. **C** HUVECs were stimulated or not with 10 ng/mL BMP10 for 2, 4, or 8 h. RT-qPCR analysis was then performed to determine mRNA expression of E2F2, CCND1 and CCNA1. Target gene expression was normalized to HPRT mRNA level using 2-ΔΔCt method and presented as relative expression (%) (BMP10-vs-vehicle) ± SEM at each time point (*n* = 3). *, #and $ represents statistical significance (BMP10-vs-NS) for CCND1, E2F2 and CCNA1, respectively. Statistical analysis was performed using Kruskal Wallis with Dunnett’s post-test^*^, ^#^, $*P* < 0.05; ^##^*P* < 0.01; ^***^ *P* < 0.001; ^****^, ^####^, ^$$$$^ *P* < 0.0001. **D** HUVECs were synchronized and stimulated as explained in panel **A**. Cell extracts were subjected to WB analysis using antibodies against pRB1-Ser807/811, CyclinD1, and P27. Quantifications were performed using HSP90. Data are presented as mean folds ± SEM of *n* = 4. *P < 0.05 using Kolmogorov–Smirnov test

Bioinformatic analysis of our phosphoproteomic results indicated that BMP9 and BMP10 stimulations may affect cell cycle in ECs (Fig. 3A and Table S5), possibly through a down-regulation of CDK4/6 and CDC7 activities, as predicted by KinSwing analysis (Fig. 3B). CDK4/6 and CDC7 are pivotal regulatory kinases involved in governing the G1/S transition during the cell cycle [28, 29]. In order to test the impact of BMP10 stimulation on the regulation of endothelial cell cycle, HUVECs were synchronized at the G1 phase via serum starvation, followed by induction of cell cycle progression through serum replenishment in the presence or absence of BMP10. Analysis of cell cycle progression was assessed using EdU (labels S-phase cells) and PI (total DNA content) staining, followed by flow cytometry analysis. The results revealed that BMP10 treatment reduced the proportion of cells in the S phase, both under low (0.5%) and high (5%) serum conditions (5.6 to 1.0% and 11.4 to 2.8%, respectively), demonstrating a significant effect of BMP10 on blocking the G1/S transition (Fig. 7A).

CDK4/6 associates with type-D cyclins (D1, D2, and D3), key regulators required for their activation. In turn, active CDK4/6 complexes phosphorylate several downstream substrates, including the tumor suppressor RB1, the protein product of the retinoblastoma tumor susceptibility gene. Hyperphosphorylation of RB1 releases E2F transcription factors, driving the expression of genes implicated in the G1/S transition (Fig. 7B) [45]. To gain deeper insights into the role of BMP10 stimulation on the G1/S transition in ECs, we studied several key effectors implicated in this process. Our data revealed that BMP10 stimulation markedly reduced the mRNA expression of *E2F2*, a CDK4/6 activity-dependent transcription factor, as early as 2 h post-stimulation compared to NS cells (Fig. 7C). Additionally, mRNA levels of *CCND1* (gene encoding cyclin D1) and *CCNA1* (gene encoding cyclin A1, a key regulator of CDK2) were also down-regulated after BMP10 stimulation of HUVECs compared to NS cells (Fig. 7C). At the protein level, BMP10 stimulation of ECs is actually accompanied by a decrease in RB1 phosphorylation and in cyclin D1 abundance (Fig. 7D). Moreover, the abundance of the CDK4/6 inhibitor P27 is increased in BMP10-stimulated cells compared to NS ones (Fig. 7D). Collectively, these results indicate that BMP10 stimulation negatively regulates the endothelial cell cycle, specifically by inhibiting the G1/S transition through a modulation in levels of several key factors in this pathway.

## Discussion

This deep phosphoproteomic study identified many cellular proteins whose phosphorylation status is modified in human endothelial cells in response to BMP9 and BMP10 stimulation. In addition to the canonical SMAD pathway, it revealed the activation of another signaling pathway involving the MEKK4/P38 axis. This pathway was responsible for the phosphorylation of HSP27 and Eps15, but also for the transcriptional regulation of *SELE*, *PTGS2* and *HAS2* expression. The activation of this pathway required a SMAD-dependent transcriptional step to induce GADD45β expression, consequently leading to the delayed activation of P38. In addition, our results pointed towards an inhibitory role of BMP9 and BMP10 stimulation on endothelial cell cycle, which was confirmed functionally by BMP10-mediated inhibition of the G1/S transition.

In this study, we analyzed both BMP9 and BMP10 signaling, as, despite sharing the same affinity to the type-I receptor ALK1, they exhibit different binding affinities to the type-II receptors (BMPRII, ActRIIA and ActrIIB) [46]. Our data clearly showed that, under a 30-min stimulation with 10 ng/mL of BMP9 or BMP10, HUVECs displayed highly similar changes in their phosphoproteomes, in accordance with a previous study revealing equivalent transcriptomic responses [47], strongly suggesting that BMP9 and BMP10 activate very similar signaling pathways in ECs. Nonetheless, these in vitro findings do not rule out specific in vivo cardiovascular roles for BMP9 and BMP10 that can arise from differences in expression patterns (localization, kinetic, accessibility), that have been described by our group [48, 49] but also many other groups [50, 51], as discussed in a recent review [2]. Our study revealed that BMP9 and BMP10 stimulation induced differential phosphorylation of ALK1 on 2 sites, Ser^155^ and Ser^161^ compared to NS cells. Both sites are located in the juxtamembrane domain, directly upstream to the GS domain in which specific sites are phosphorylated by the type II receptor [3, 52]. Interestingly, phosphorylation of Ser^165^, Ser^172^ and Thr^176^, located within the juxtamembrane domain of ALK5, have been previously described as not to be involved in SMAD canonical signaling but rather important for some TGF-β1-mediated functions, such as cell proliferation [53, 54]. Accordingly, our results support that ALK1 juxtamembrane sites do not alter the canonical SMAD pathway. These sites may therefore contribute to other BMP9 and BMP10-mediated functional outcomes.

**Fig. 8:**
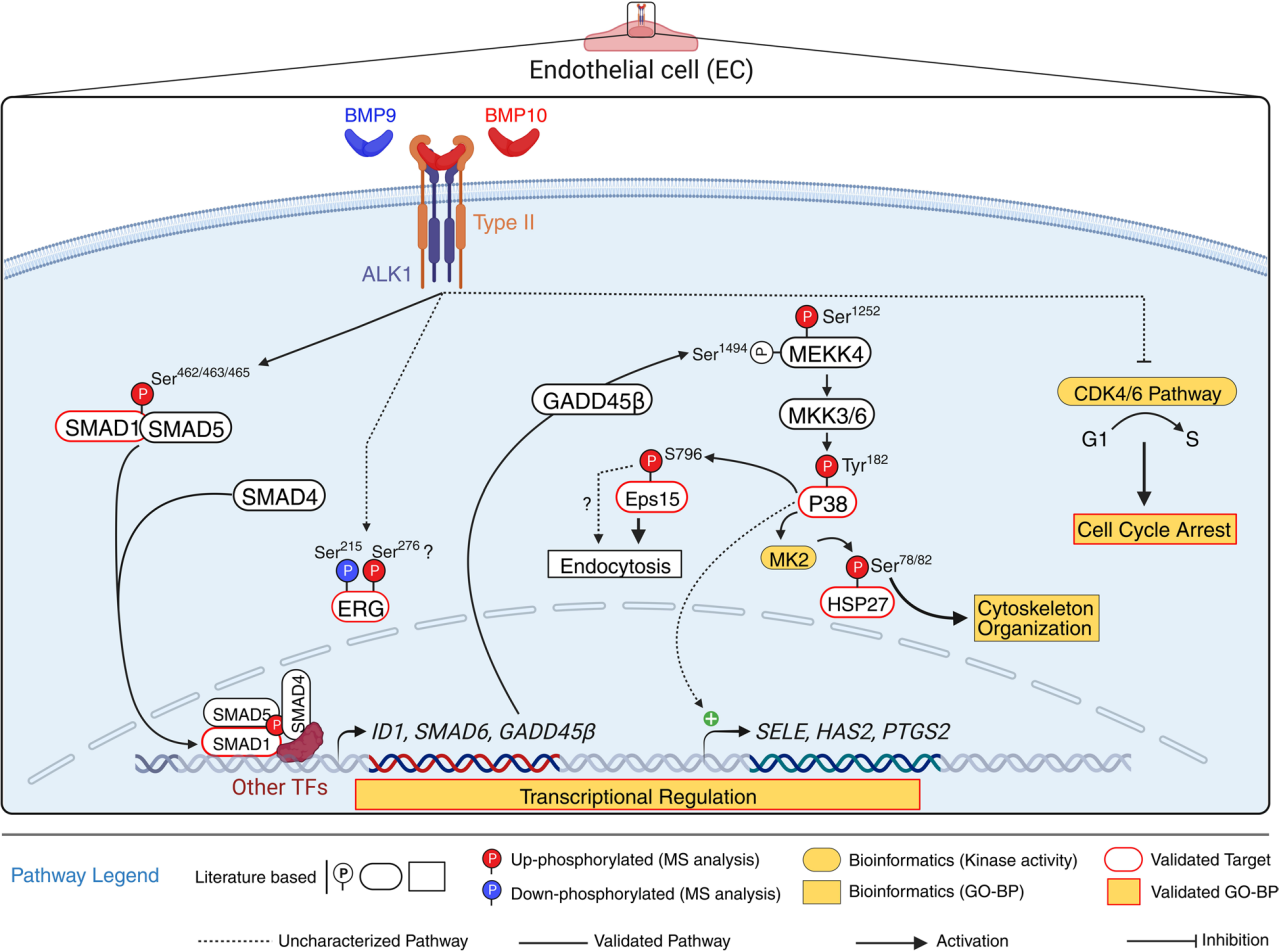
Working model: BMP9/BMP10/ALK1/SMAD4 Signaling drives the regulation of direct and indirect pathways in ECs. Binding of BMP9 and BMP10 (BMP9/10) to ALK1 along with a type II receptor on ECs mediates the activation of ALK1, leading to the initiation of direct and indirect pathways. The direct pathway involves the SMAD cascade, where activated ALK1 phosphorylates the C-terminus of SMAD1 and SMAD5, allowing the recruitment of SMAD4, forming a trimeric SMAD complex. This trimeric SMAD complex subsequently translocated to the nucleus, where it binds to the promoters of target genes with the assistance of other transcription factors (TFs), thereby regulating their expression levels. Among these, BMP9/10 induce the expression of ID1, SMAD6 and GADD45β (newly identified target). The indirect pathway involves the expression of GADD45β, an activator of MEKK4, which mediates activation of P38/MK2 signaling axis by these ligands. In this cascade, P38 phosphorylates Eps15-Ser796, while P38/MK2 phosphorylates HSP27-Ser78/82, which have been described to play important roles in endocytosis and cytoskeleton organization, respectively. BMP9 and BMP10 also induce the differential phosphorylation of the transcription factor ERG via an uncharacterized mechanism. Additionally, P38 activation regulates a subset of BMP9/10-induced genes, including SELE, HAS2, and PTGS2. On the other hand, BMP9/10 downregulates the CDK4/6 pathway leading to inhibition of G1/S transition and cell cycle arrest

We showed that BMP9 and BMP10 stimulations activate the P38 MAPK pathway in ECs, peaking one hour after BMP addition, and therefore supporting an indirect mechanism. Our results demonstrate that SMAD-mediated GADD45β expression is required for this delayed activation. The GADD45 family of proteins (α, β, and γ) are critical stress sensors that mediate various cellular responses, including DNA repair, cell cycle arrest, and apoptosis [55]. GADD45 α, β, γ proteins are differentially induced and can interact with different proteins (CDK1, MTK1/MEKK4, p21, p38 and PCNA) [56] and mediate specific cellular processes. TGF-ß has been shown to specifically induce GADD45β expression [42, 57]. Much less is known concerning GADD45 regulation by BMPs. To our knowledge, only one publication showed that BMP2 could induce *GADD45β* mRNA expression and this was in chondrocytes [58]. Interestingly, binding of GADD45β to MEKK4 N-terminus has been shown to induce MEKK4 dimerization, allowing its trans-autophosphorylation at Thr^1493/1494^ in its kinase activation loop, leading to its activation [43] and thus P38 activation [42]. Of note, our phosphoproteomic analysis identified MEKK4 to be differentially phosphorylated after BMP9 and BMP10 stimulations at Ser^1252^, but this site is different from the previously observed ones (MEKK4-Thr^1493/1494^). This GADD45β/P38 pathway has been shown to induce TGF-β-induced Biglycan expression [59]. We demonstrated that this P38 signaling cascade can then activate MK2, which subsequently phosphorylates HSP27-Ser^78/82^. Our data also show that P38 is implicated in the transcriptional regulation of the expression of several genes after stimulation of ECs with BMP10 (*SELE*, *HAS2*, and *PTGS2*), supporting the hypothesis that BMP10 stimulation may regulate gene expression through at least two different ways: direct SMAD-dependent transcription (*ID1*, *SMAD6*) and SMAD-dependent P38-activated transcription (*SELE*, *HAS2*, and *PTGS2*). Together our results allowed to propose the BMP9/BMP10 signaling working model presented in Fig. 8.

We found that BMP9 and BMP10 stimulations induce the phosphorylation of Eps15-Ser^796^. This site has previously been described as phosphorylated in response to TNFα, EGF, and IL-1β [39]. However, the molecular mechanism dependent on this phosphorylation event remains unknown. It has been shown that Eps15, along with its homologous protein Eps15R, act as scaffold proteins and play important roles in endocytosis [60]. Interestingly, Ser^796^ is located between Grb2/AP2 binding domains that are involved in endocytosis of many receptors, including EGFR [34, 61], and the C-terminal ubiquitin-binding domains (UIMs) [60], suggesting a role in clathrin-mediated endocytosis. A direct interaction between Eps15R and BMPRII has been previously described [62], and Eps15R interaction with SMAD1 was found to be required for BMP signaling in Xenopus animal caps [63]. Together, these results suggest a functional link between Eps15R and BMP-induced pathways. Due to the similarity between the functional domains of Eps15 and Eps15R, these studies suggest that Eps15 might also interact with BMP receptors and contribute to their endocytosis and/or downstream signaling in ECs.

Our phosphoproteomic analysis revealed that both BMP9 and BMP10 stimulations induced the phosphorylation of HSP27 in ECs. HSP27 phosphorylation has been shown to be implicated in many biological processes including apoptosis, proliferation, migration, differentiation, transcriptional regulation and cytoskeletal organization [64–66]. In ECs, HSP27 plays important roles in the regulation of actin filament remodeling and EC barrier function [67–70]. It was recently shown that BMP2- and BMP6-induced HSP27 phosphorylation via P38 activation promoted endothelial cell migration [71]. Although the targeted HSP27 phosphorylation site was not mentioned in this study, the time of stimulation used (45 min) is in accordance with the phosphorylation kinetics of P38 and HSP27 observed in response to BMP10 in our work, suggesting that other BMPs could activate this P38/HSP27 pathway in a similar manner. Our study also identified LIMK1-Ser^310^ phosphorylation to be down-regulated in response to BMP9 and BMP10 (Table S4). LIMK1 regulates actin dynamics by phosphorylating cofilin, which leads to actin depolymerization. LIMK1 has been shown to interact with BMPRII [72] and endoglin [73], and the interaction between LIMK1 and BMPRII inhibited LIMK1's ability to phosphorylate cofilin, which could then be alleviated by the addition of BMP4 [72]. While it was also shown that VEGF induces LIMK1 phosphorylation at Ser^310/323^ via MKK4/P38/MK2 axis in HUVECS, only LIMK1-Ser^323^ was involved in VEGF-induced actin remodeling and cell migration [74]. LIMK1 phosphorylation might thus be an interesting hit to be further studied, given the role of actin turnover in cell migration and the involvement of cell migration in the development of HHT arteriovenous malformations (AVMs) [75].

Our work also revealed that BMP9 and BMP10 treatment induce the differential phosphorylation of the key endothelial transcription factor ERG. ERG has been described as a key regulator of vascular homeostasis, being implicated in vascular development and angiogenesis, arterial specification, vascular homeostasis, vessel stability, and permeability [36]. We were able to validate that BMP9 and BMP10 stimulations of ECs induce a global ERG-Serine phosphorylation, while decreasing specifically ERG-Ser^215^ phosphorylation. It was previously demonstrated that ERK2 phosphorylates ERG at Ser^96/215/276^, but that only Ser^215^ was required for ERG activity in prostate cancer cells [37], and that this phosphorylation site was important for Dll4 expression in ECs [38]. Interestingly, we found that BMP10 stimulation inhibited both basal and VEGF-induced ERG-Ser^215^ phosphorylation. Of note, it was shown that ERG silencing caused a reduction in ALK1/endoglin signaling in HUVECs by regulating their expressions [76]. Given the critical roles played by ERG in ECs, it represents a target of choice for future studies.

Finally, this work highlighted the role played by BMP9 and BMP10 stimulations in the inhibition of EC cycle, which is in accordance with the known role of BMP9 and BMP10 in vascular quiescence [2]. However, the molecular mechanism leading to inhibition of EC proliferation by BMP9 or BMP10 remains poorly understood. We highlighted that anti-proliferative signals were detectable after only 30 min stimulation with BMP9 or BMP10. It was already demonstrated that BMP9 suppresses proliferation of human aortic endothelial cells by modulating various cell cycle-related proteins, notably P27 and cyclin D1 [77]. This inhibition was shown to be SMAD1/5-dependent and required the expression of the CDK4/6 inhibitor P27. In our study, we found that BMP10 stimulation rapidly reduces the mRNA expression of three cell cycle regulatory genes (*CCND1*, *CCNA1*, and *E2F2*), while a decrease of RB1 phosphorylation and cyclin D1 protein expression was clearly detected after 18 h of stimulation. In vivo, *Bmp9*-KO mice showed an increase in liver EC proliferation, and the upregulation of several genes involved in cell cycle such as Ccne1, *Pttg1* and *E2f2* (Desroches-Castan et al., Cardiovas Research, In press). Interestingly, arteriovenous malformations (AVMs) have recently been attributed to an excess of EC proliferation, which could be reduced by the injection of Palbociclib, a CDK4/6 inhibitor [78, 79]. Signs of abnormal cell cycle progression were also observed in HHT patient skin biopsies [35, 80]. Therefore, CDK4/6 inhibitors could be potentially repurposed in HHT. It will also be interesting in the future to test the potential links between the inhibitory role of BMP19 and BMP10 in cell cycle regulation and the induction of GADD45β, which has been involved in cell cycle control [81].

## Conclusions

In conclusion, our study revealed numerous differentially regulated phosphosites after a 30-min stimulation of HUVECs with BMP9 and BMP10. While we could validate several important hits and link them to specific signaling pathways, we could not study all of them. However, we hope that the vascular biology community will benefit from this unique high-throughput phosphoproteomic study of BMP9 and BMP10-mediated signaling to further characterize the role of these 2 ligands in physiological conditions but also in pathological vascular diseases, such as HHT and PAH.

### Supplementary Information

**Additional file 1.**

## Data Availability

The datasets generated and analysed during the current study are available in the repository ProteomeXchange Consortium via the PRIDE partner repository [82] with the dataset identifier PXD044952.

## Abbreviations

ActRII: Activin receptor type II
ALK1: Activin receptor-like kinase 1
BMP: Bone morphogenetic protein
BMPRII: BMP receptor type II
CDK: Cyclin-dependent kinases
GDF: Growth and differentiation factors
GS: Glycine/serine
HHT: Hereditary hemorrhagic telangiectasia
EC: Endothelial cells
ERK: Extracellular signal-regulated kinases
HPAH: Heritable pulmonary arterial hypertension
Eps15: EGF receptor pathway substrate
ERG: ETS Transcription Factor ERG
GADD45β: Growth arrest and DNA damage inducible beta
HSP27: Heat shock protein
HUVEC: Human Umbilical Vein Endothelial Cells
MAPK: Mitogen-activated protein kinases
SMAD: SMA ("small" worm phenotype) and MAD family ("Mothers Against Decapentaplegic")
TGF-β: Transforming growth factor-beta
