# Supplementary material for: Large-scale phosphoproteomics reveals activation of the MAPK/GADD45β/P38 axis and cell cycle inhibition in response to BMP9 and BMP10 stimulation in endothelial cells
Source: Cell Commun Signal. 2024 Mar 4;22:158. doi: 10.1186/s12964-024-01486-0 (PMC10910747; doi:10.1186/s12964-024-01486-0)
Supplement: Supplementary file 1 — Additional file 1. [file 12964_2024_1486_MOESM1_ESM.zip › 12964_2024_1486_MOESM1_ESM Final version corrected.docx]

**SUPPLEMENTAL INFORMATION**This section includes Figures S1–S6 and the description of supplementary tables S1-S5 (provided as separate excel files)

**Supplemental Figures**


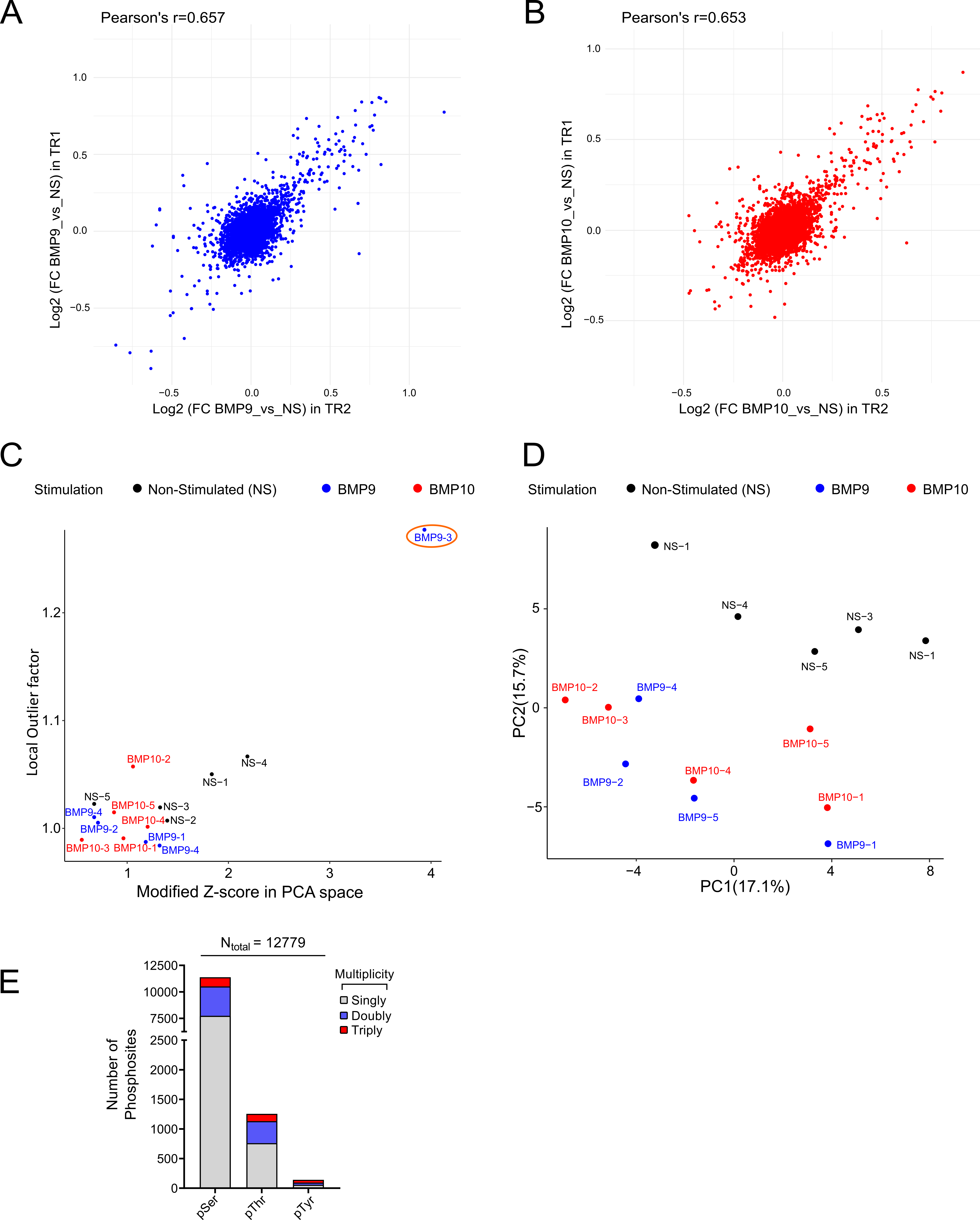


**Fig. S1. Quality control of Phosphoproteomic data**

**(A and B)** Scatter plots comparing log2 fold change values of phosphosites between technical replicate 1 (TR1, y-axis) and technical replicate 2 (TR2, x-axis), for samples treated by BMP9 (A) and BMP10 (B). Pearson’s correlation coefficient (r) is reported.

**(C)** Identification of sample BMP9-3 (highlighted in orange) as an outlier by measuring local outlier factor (LOF) (y-axis) and modified Z-score (x-axis).

**(D)** Principal component analysis (PCA) of phosphoproteomic data after quality control correction.

**(E)** Distribution of the 12779 quantified phosphosites across all samples according to their phosphorylation residue and multiplicity.


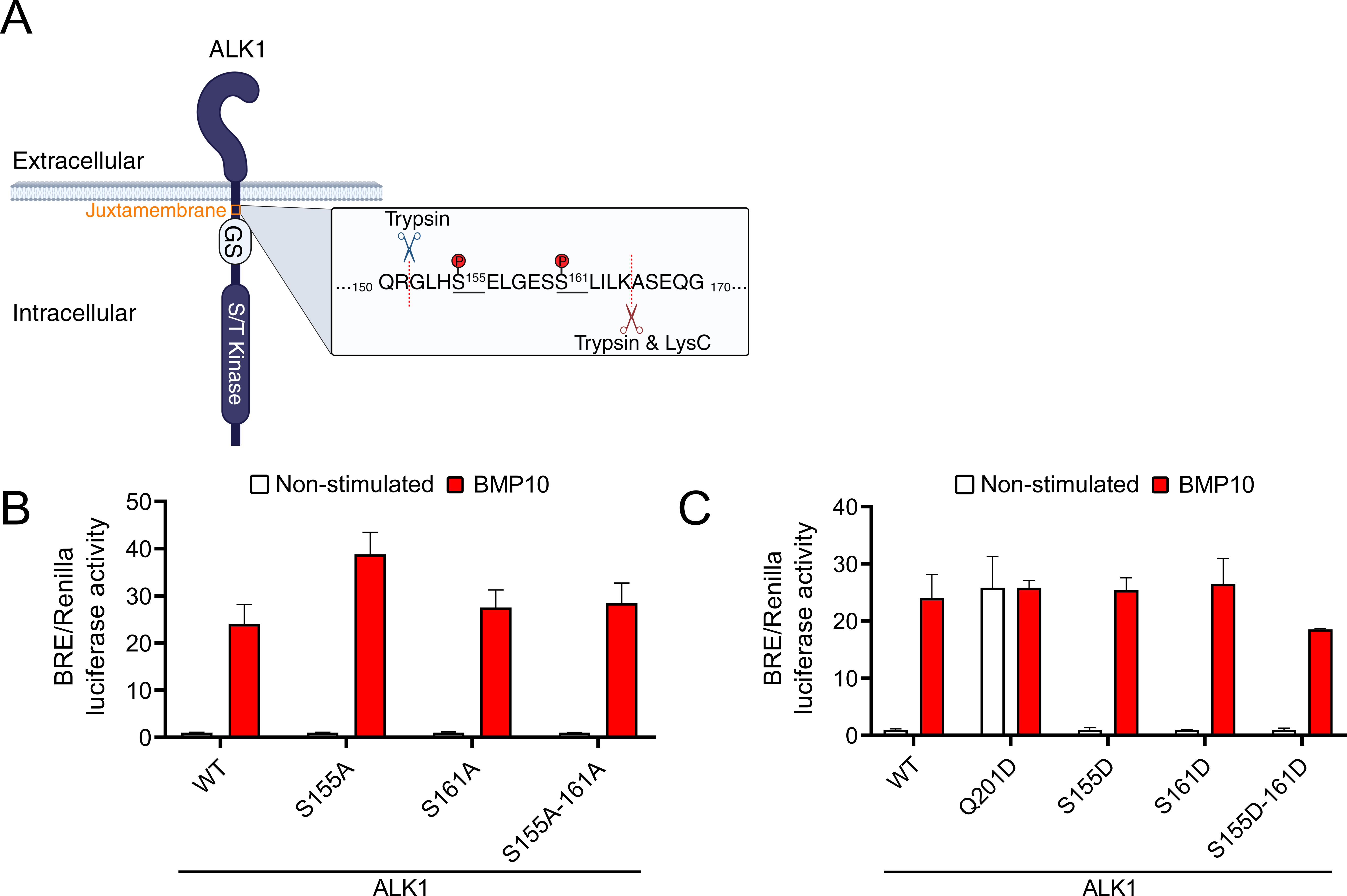


**Figure S2. Functional testing of the ALK1 juxtamembrane phosphorylations Ser^155^ and Ser^161^ in response to BMP10 stimulation using the ID1-BRE-Luciferase assay**

(**A**) Structural overview of the extracellular and intracellular domains of the type I receptor ALK1. The intracellular domain comprises the GS domain and the Ser/Thr kinase domain. The juxtamembrane region (highlighted in orange) located directly upstream of the GS domain bears the phosphopeptide obtained by phosphoproteomic analysis after digestion. This peptide highlights Ser^155^ and Ser^161^ phosphosites which were found differentially up-phosphorylated by BMP9 and BMP10.

(**B and C**) Relative BRE (BMP Response Element) luciferase activity measured in NIH-3T3 cells overexpressing wild-type (WT) or indicated ALK1 mutants. Serine residues were mutated to either alanine (S>A; phosphodead) (panel B) or aspartic acid (S>D ; phosphomimetic) (panel C). ALK1Q201D represents a positive control for constitutively active ALK1. Of note, in absence of ALK1, BMP10 stimulation did not lead to any luciferase signal (data not shown). BRE firefly luciferase activities were normalized to renilla luciferase activity. Data shown represent the mean ± SEM of at least two independent experiments.


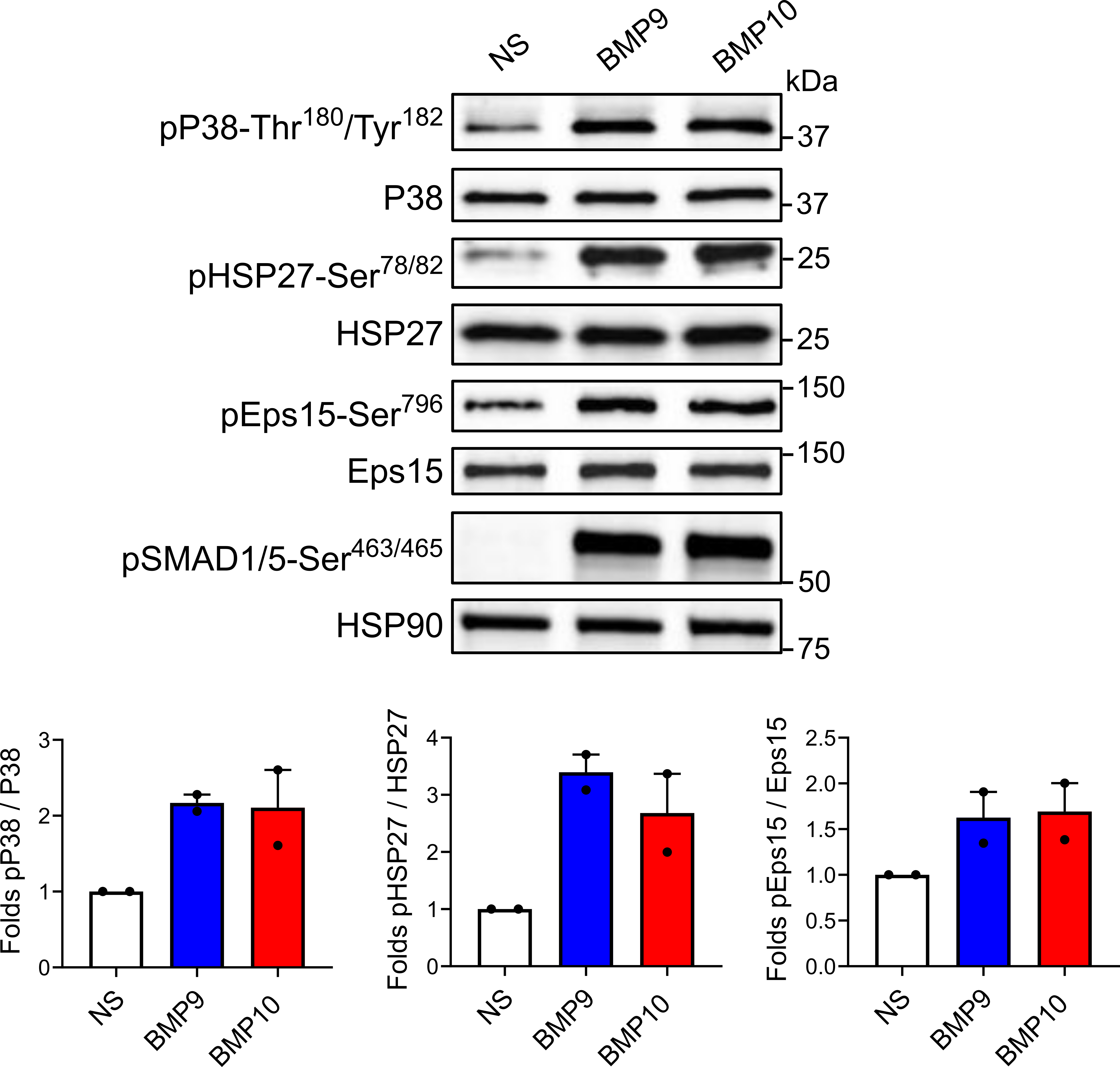


**Figure S3. BMP9 and BMP10 induce P38, HSP27 and Eps15 phosphorylations in HUVECs**

Cells were not stimulated (NS) or stimulated with 10ng/mL BMP9 or BMP10 for 30 min. Cell extracts were subjected to western blotting (WB) analysis using antibodies against phosphorylated (p) P38-Thr^180^/Tyr^182^, P38, pHSP27-Ser^78/82^, HSP27, pEps15-Ser^796^, Eps15, pSMAD1/5-Ser^463/465^ and HSP90 (loading control for pSMAD1/5). Quantification of phosphorylation for P38, HSP27 and Eps15 was normalized to their respective total protein content. Data are presented as mean fold change (BMP9-vs-NS or BMP10-vs-NS) ± SEM of n=2 independent experiments.


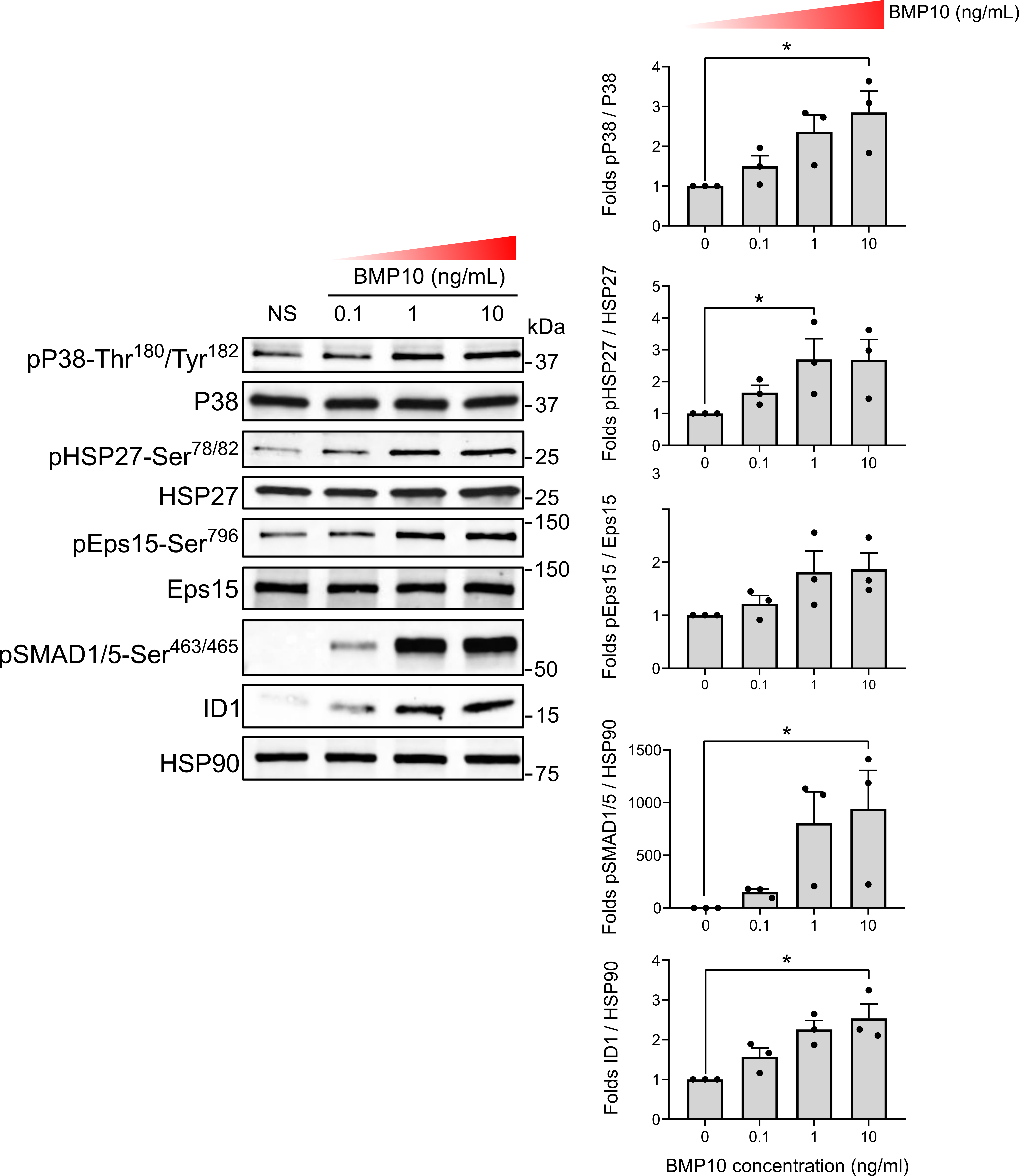


**Figure S4. BMP10 induces P38, HSP27 and Eps15 phosphorylations in a dose-dependent manner in HUVECs**

Cells were non-stimulated (NS) or stimulated with different doses of BMP10 (0.1, 1 or 10ng/mL) for 30 min. Cell extracts were subjected to WB analysis using antibodies against phosphorylated (p) P38-Thr^180^/Tyr^182^, P38, pHSP27-Ser^78/82^, HSP27, pEps15-Ser^796^, Eps15, pSMAD1/5-Ser^463/465^, ID1 and HSP90 (loading control). Quantification of phosphorylation for P38, HSP27 and Eps15 was normalized to their respective total protein content, except for pSMAD1/5 and ID1, which were normalized to HSP90. Data are presented as mean fold change (BMP10-vs-NS) ± SEM of n=3 independent experiments. Statistical analysis was performed using Kruskal Wallis with Dunn’s multiple comparisons post-test. * P < 0.05.


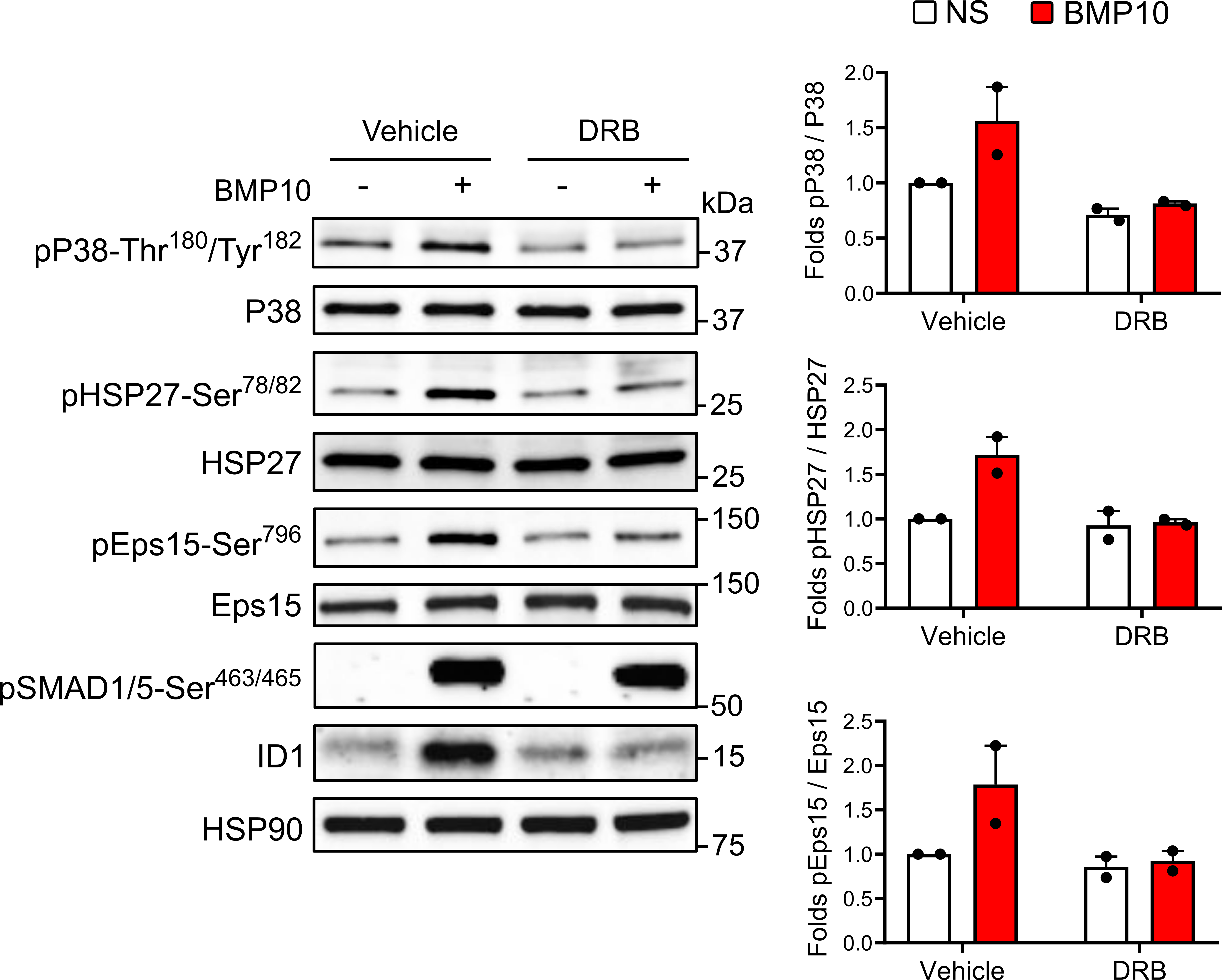


**Figure S5. BMP10-induced P38, HSP27 and Eps15 phosphorylations are inhibited by the transcription inhibitor DRB**

HUVECs were pre-treated either with vehicle or the transcription inhibitor DRB (5,6-Dichlorobenzimidazole 1-β-D-ribofuranoside) (100 μM) for 30 min, then stimulated with 10ng/mL BMP10 or not (NS) for another 30 min. Cell extracts were subjected to WB analysis using antibodies against phosphorylated (p) P38-Thr^180^/Tyr^182^, P38, pHSP27-Ser^78/82^, HSP27, pEps15-Ser^796^, Eps15, pSMAD1/5-Ser^463/465^, ID1 and HSP90 (loading control for pSMAD1/5 and ID1). Quantification of phosphorylation for P38, HSP27 and Eps15 was normalized to their respective total protein content. Data are presented as mean fold change (BMP10-vs-NS) ± SEM of n=2 independent experiments.


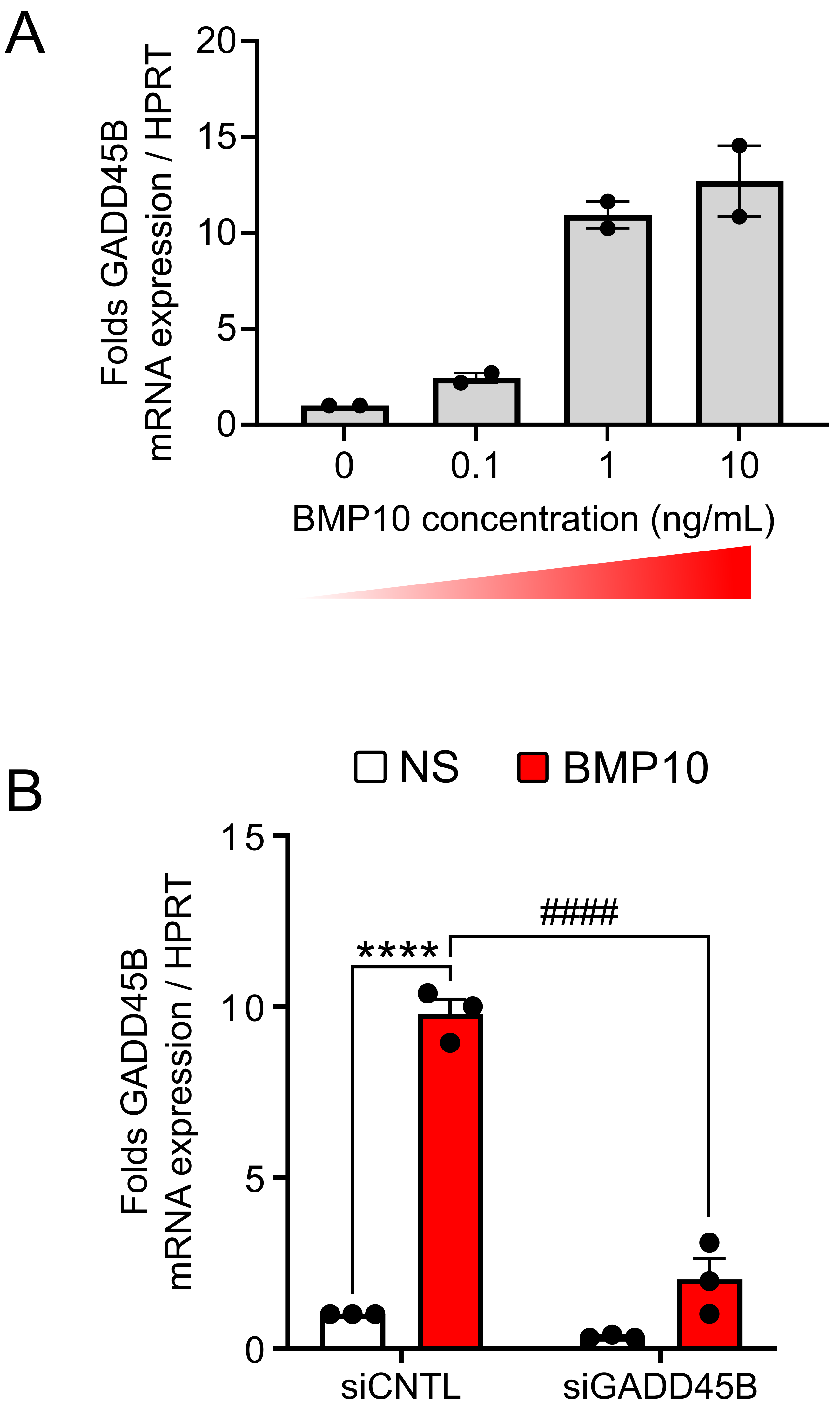


**Figure S6. *GADD45β* mRNA expression in response to BMP10 stimulation and/or siRNA GADD45β inhibition**

(**A**) HUVECs were non-stimulated (0) or stimulated with different doses of BMP10 (0.1, 1 or 10 ng/mL) for 30 min. RT-qPCR analysis was then performed to determine mRNA expression of *GADD45β*. Target gene expression was normalized to *HPRT* mRNA levels using 2^-ΔΔCt^ method and presented as fold induction (BMP10-vs-vehicle) ± SEM of n=2 independent experiments.

(**B**) HUVECs were treated either with scrambled siRNA (siCTL) or siRNA against GADD45β (siGADD45β) for 48 hours, followed by stimulation with 10ng/mL BMP10 or not (NS) for 30 min. RT-qPCR analysis was then performed to determine mRNA expression of *GADD45β*. Target gene expression was normalized to *HPRT* mRNA level using 2^-ΔΔCt^ and presented as fold induction (BMP10-vs-NS) ± SEM of n=3 independent experiments. Statistical analysis was performed using two-way ANOVA followed by Sidak's multiple comparisons post-test. ****^,^ ^####^ P < 0.0001. *: BMP10-vs-NS; ^#^: siCTL-vs- siGADD45β.

**Supplemental Tables**

**Table S1**. List of primers used for ALK1 site-directed mutagenesis and RT-qPCR, related to STAR Methods. Sheet 1: List of primers used for the generation of mutant ALK1 constructs by site-directed mutagenesis-associated PCR designed using the QuickChange Primer Design Program (Agilent). Sheet 2: List of forward and reverse primers used for quantitative RT-qPCR designed using Primer-Blast on GenBank sequences. All listed primer pairs are separated by at least one intron on the corresponding genomic DNA or span an exon-exon junction.

**Table S2**. Key resources table.

**Table S3**. Phosphoproteome and Proteome identified in HUVECs stimulated or not with BMP9 or BMP10, related to Figures 1 and 2. Sheet 1: Phosphoproteomic data combined from technical replicates 1 and 2. Sheets 2 and 3: Phosphoproteomic data from technical replicates 1 and 2, respectively. Sheet 4: Global proteomic analysis prior to phosphoenrichment.

**Table S4**. List of kinases and phosphatases differentially phosphorylated by BMP9 and/or BMP10 from phosphoproteomics data extracted from Table S3.

**Table S5**. Bioinformatic analyses of the phosphoproteomic changes in response to BMP9 and BMP10 in HUVECs, related to Figure 3. Sheets 1 and 2: Gene-ontology on biological processes (GO-BP) of genes encoding differentially phosphorylated proteins by BMP9 and BMP10, respectively. Sheets 3 and 4: WikiPathways analysis of genes encoding differentially phosphorylated proteins by BMP9 and BMP10, respectively. Sheets 5 and 6: Tables of Kinase activities analysis using KinSwing tool of phosphosites regulated by BMP9 and BMP10, respectively. Positive swing scores indicate predicted activation of a kinase, while negative scores indicate predicted under-activation of a kinase. Sheet 7: Kinase-target relationships extracted from KinSwing analysis. Sheets 8 and 9: Post-translational modification signature enrichment analysis (PTM-SEA) of phosphosites obtained from BMP9-vs-NS and BMP10-vs-NS data, respectively. PERT, Perturbation; PSP, Phosphositeplus, PATH, Pathway. Each row represents a signature (kinase, perturbation or pathway). Sheet 10: Signature-target relationships extracted from PTM-SEA analysis.
