# Supplementary material for: Large-scale phosphoproteomics reveals activation of the MAPK/GADD45β/P38 axis and cell cycle inhibition in response to BMP9 and BMP10 stimulation in endothelial cells
Source: Cell Commun Signal. 2024 Mar 4;22:158. doi: 10.1186/s12964-024-01486-0 (PMC10910747; doi:10.1186/s12964-024-01486-0)
Supplement: Supplementary file 1 — Additional file 1. [file 12964_2024_1486_MOESM1_ESM.zip › Table S2.docx]

| REAGENT or RESOURCE  Table S2 : Key ressource table | SOURCE | IDENTIFIER |
| --- | --- | --- |
| Antibodies | | |
| Phospho-SMAD1/5 (Ser463/465) (41D10) rabbit monoclonal antibody | Cell Signaling Technology | Cat# 9516  RRID:AB_491015 |
| Phospho-p38 MAPK (Thr180/Tyr182) (D3F9) rabbit monoclonal antibody | Cell Signaling Technology | Cat# 4511  RRID:AB_2139682 |
| p38 MAPK rabbit polyclonal antibody | Cell Signaling Technology | Cat# 9212 RRID:AB_330713 |
| Phospho-Eps15 (Ser796) rabbit monoclonal antibody | Dr. Hiroaki Sakurai [39] | RRID: AB_3073931 |
| Eps15 mouse monoclonal antibody | Santa Cruz | Cat# sc-390259  RRID: AB_3073929 |
| Phospho-HSP27 (Ser78/82) rabbit monoclonal antibody | R&D Systems | Cat# MAB23141  RRID: AB_3073930 |
| HSP27 (D6W5V) rabbit monoclonal antibody | Cell Signaling Technology | Cat# 95357  RRID:AB_2800246 |
| ID1 (B-8) mouse monoclonal antibody | Santa Cruz | Cat# sc-133104 RRID:AB_2122863 |
| HSP90 (C45G5) rabbit monoclonal antibody | Cell Signaling Technology | Cat# 4877  RRID:AB_2233307 |
| SMAD4 rabbit polyclonal antibody | Cell Signaling Technology | Cat# 9515  RRID:AB_2193344 |
| ERG (A7L1G) rabbit monoclonal antibody | Cell Signaling Technology | Cat# 97249  RRID:AB_2721841 |
| Phospho-ERG (Ser215) rabbit monoclonal antibody | Dr. Peter Hollenhorst [37] | RRID: AB_3073932 |
| Phospho-Serine (pSer) (PSR-45) mouse monoclonal antibody | Sigma | Cat# P5747  RRID:AB_477376 |
| Cyclin D1 (92G2) rabbit monoclonal antibody | Cell Signaling Technology | Cat# 2978  RRID:AB_2259616 |
| P27 Kip1 (D69C12) rabbit monoclonal antibody | Cell Signaling Technology | Cat# 3686  RRID:AB_2077850 |
| Phospho-Rb (Ser807/811) (D20B12) rabbit monoclonal antibody | Cell Signaling Technology | Cat# 8516  RRID:AB_11178658 |
| Phospho-ERK1/2 (Thr202/Tyr204) rabbit polyclonal antibody | Promega | Cat# V803A |
| ERK1/2 rabbit polyclonal antibody | Sigma | Cat# M5670  RRID:AB_477216 |
| Chemicals, peptides, and recombinant proteins | | |
| Recombinant Human BMP-9 Protein | R&D Systems | Cat# 3209-BP |
| Recombinant Human BMP-10 Protein | R&D Systems | Cat# 2926-BP |
| Protease inhibitor cocktail | Sigma | Cat# P8340 |
| Phosphatase inhibitor cocktail 2 | Sigma | Cat# P5726 |
| Phosphatase inhibitor cocktail 3 | Sigma | Cat# P0044 |
| LDN193189 | Sigma | Cat# SML0559 |
| SB203580 | Tocris Bioscience | Cat# 1202 |
| PF3644022 | Tocris Bioscience | Cat# 4279 |
| Actinomycin D | Sigma | Cat# A1410 |
| DRB (5, 6-Dichlorobenzimidazole 1-β-D-ribofuranoside) | Sigma | Cat# D1916 |
| rLys-C, Mass Spec Grade | Promega | Cat# V1671 |
| sequencing grade-modified trypsin | Promega | Cat# V5111 |
| TMTpro 16-plex Label Reagent Set | ThermoFisher Scientific | Cat# A44520 |
| Critical commercial assays | | |
| Twinlite Firefly and Renilla Luciferase Reporter Gene Assay System | Perkin Elmer | Cat# 6016799 |
| Deposited data | | |
| Mass spectrometry proteomic and phosphoproteomic data | This paper | PRIDE: <https://www.ebi.ac.uk/pride/archive/> Identifier: PXD044952 |
| Experimental models: Cell lines | | |
| Human umbilical vein endothelial cells (HUVECs) | Lonza | C2517A |
| Murine NIH-3T3 fibroblasts | ATCC | CRL-1658^TM^ |
| Oligonucleotides | | |
| RT-qPCR primers | Sigma | Table S1 |
| Mutagenesis primers for ALK1 mutations | Sigma | Table S1 |
| Recombinant DNA | | |
| ALK1 plasmids cloned in pcDNA3.1 | Thermo Fisher Scientific | V79020 |
| BRE firefly luciferase from Korchynskyi and ten Dijke P. | [Korchynskyi and ten Dijke P. 2002](https://pubmed.ncbi.nlm.nih.gov/11729207/), Promega | E1751 |
| Renilla luciferase | Promega | E2241 |
| Software and algorithms | | |
| KinSwingR v1.16.0 | Engholm-Keller et al. 2019 [18] | <https://bioconductor.org/packages/release/bioc/html/KinSwingR.html> |
| PTMsigDB; obtained from WebGestaltR v0.4.6 | Krug et al. 2019 [20] | <https://www.gsea-msigdb.org/gsea/msigdb/ptmsig_genesets.jsp> |
| R Statistical Computing Software v4.2.1 | R Development Core Team. | <https://www.R-project.org/> |
| GraphPad Prism software v8.2.1 | GraphPad Software | <https://www.graphpad.com/> |
| Metascape | Zhou et al. 2019 [17] | <https://metascape.org/> |
| MaxQuant v1.6.17.0 | Max Planck Institute of Biochemistry | <https://www.maxquant.org/> |
| Xcalibur v2.9 | Thermo Fisher Scientific | <https://www.thermofisher.com/fr/fr/home/industrial/mass-spectrometry/liquid-chromatography-mass-spectrometry-lc-ms/lc-ms-software/lc-ms-data-acquisition-software/xcalibur-data-acquisition-interpretation-software.html> |
| Prostar | [Wieczorek et al. 2016](https://pubmed.ncbi.nlm.nih.gov/27605098/) | <https://www.prostar-proteomics.org/> |
| ClusterProfiler v4.6.2 | [Wu et al. 2021](https://pubmed.ncbi.nlm.nih.gov/34557778/) | <https://bioconductor.org/packages/release/bioc/html/clusterProfiler.html> |
